# Lectin-Glycan Interaction Network-Based Identification of Host Receptors of Microbial Pathogenic Adhesins

**DOI:** 10.1128/mBio.00584-16

**Published:** 2016-07-12

**Authors:** Francesco S. Ielasi, Mitchel Alioscha-Perez, Dagmara Donohue, Sandra Claes, Hichem Sahli, Dominique Schols, Ronnie G. Willaert

**Affiliations:** a Department of Bioengineering Sciences, Structural Biology Brussels, International Joint Research Group VUB-EPFL BioNanotechnology & NanoMedicine (NANO), Vrije Universiteit Brussel, Brussels, Belgium; b Department of Electronics and Informatics (ETRO), AVSP Lab, International Joint Research Group VUB-EPFL BioNanotechnology and NanoMedicine (NANO), Vrije Universiteit Brussel, Brussels, Belgium; c Department of Microbiology and Immunology, Rega Institute for Medical Research, KU Leuven, Leuven, Belgium; d Interuniversity Microelectronics Centre (IMEC), Leuven, Belgium

## Abstract

The first step in the infection of humans by microbial pathogens is their adherence to host tissue cells, which is frequently based on the binding of carbohydrate-binding proteins (lectin-like adhesins) to human cell receptors that expose glycans. In only a few cases have the human receptors of pathogenic adhesins been described. A novel strategy—based on the construction of a lectin-glycan interaction (LGI) network—to identify the potential human binding receptors for pathogenic adhesins with lectin activity was developed. The new approach is based on linking glycan array screening results of these adhesins to a human glycoprotein database via the construction of an LGI network. This strategy was used to detect human receptors for virulent *Escherichia coli* (FimH adhesin), and the fungal pathogens *Candida albicans* (Als1p and Als3p adhesins) and *C. glabrata* (Epa1, Epa6, and Epa7 adhesins), which cause candidiasis. This LGI network strategy allows the profiling of potential adhesin binding receptors in the host with prioritization, based on experimental binding data, of the most relevant interactions. New potential targets for the selected adhesins were predicted and experimentally confirmed. This methodology was also used to predict lectin interactions with envelope glycoproteins of human-pathogenic viruses. It was shown that this strategy was successful in revealing that the FimH adhesin has anti-HIV activity.

## IMPORTANCE

Microbial pathogens may express a wide range of carbohydrate-specific adhesion proteins that mediate adherence to host tissues. Pathogen attachment to host cells is achieved through the binding of these lectin-like adhesins to glycans on human glycoproteins. In only a few cases have the human receptors of pathogenic adhesins been described. We developed a new strategy to predict these interacting receptors. Therefore, we developed a novel LGI network that would allow the mapping of potential adhesin binding receptors in the host with prioritization, based on the experimental binding data, of the most relevant interactions. New potential targets for the selected adhesins (bacterial uroepithelial FimH from *E. coli* and fungal Epa and Als adhesins from *C. glabrata* and *C. albicans*) were predicted and experimentally confirmed. This methodology was also used to predict lectin interactions with human-pathogenic viruses and to discover whether FimH adhesin has anti-HIV activity.

## INTRODUCTION

Adherence of pathogenic microbes to host tissues can occur at different sites in the human body. In the case of epithelial and endothelial tissues, one of the potential adhesion targets is represented by the glycocalyx, i.e., the extracellular mesh of carbohydrate-rich molecules bound to the cell membranes or secreted by cells into the external medium (1). Microbial adhesion to components of the glycocalyx, such as glycosylated host receptors or other glycoproteins, is often mediated by adhesion proteins endowed with lectin activity (2, 3). These lectin-like adhesins, expressed on the microbial surface, recognize the highest-affinity specific glycan regions on the binding receptors, conventionally referred to as the “glycan determinants.” The typical glycan determinant includes two to six linearly arranged monosaccharides plus their branching residues or modifications (phosphorylation, sulfation, acetylation), which may be accommodated by the adhesin binding pocket too (4). A qualitative and semiquantitative analysis of the specificity of a lectin for glycan determinants can be performed by glycan array screening (5).

Urinary tract infections (UTIs) caused by uropathogenic *Escherichia coli* (UPEC) are some of the best-studied bacterial pathogen infections (6). Adherence to host cells is mediated by type 1 fimbriae, which are protein structures expressed on the bacterial cell surface (7, 8). The amino-terminal lectin domain (LD) of the fimbrial FimH subunit (FimH-LD) binds specifically to mannose and mannose-containing oligosaccharides on host uroepithelial cells (9–11). The N-glycan core structure Man-β-1,4-GlcNAc-β-1,4-GlcNAc-β is the preferential binding receptor for FimH-LD. Fimbriated *E. coli* expressing FimH is able to bind uroplakins Ia and Ib, which are two glycoproteins of the apical urothelial plaques carrying high-mannose glycans (12) and the main urothelial receptors for type 1 fimbriae (13).

Candidiasis is a fungal infection caused by the adhesion of *Candida* yeast species to host cells. *Candida albicans* and *C. glabrata* are commensal yeasts of the human gastrointestinal tract, but they are also the major causes of opportunistic *Candida* infections in susceptible hosts (14, 15). The Als (agglutinin-like sequence) family is the best-characterized adhesin family of *C. albicans* (16). The binding of Als proteins to human epithelial tissues has been attributed to the N-terminal part of the protein, which contains tandem immunoglobulin-like domains that are able to adhere to host proteins (17, 18). Among the best-studied Als proteins are Als1p and Als3p, both of which are responsible for the mediation of cellular adhesion to a broad range of ligands, such as fibronectin (FN), laminin, and collagen IV, as well as fibrinogen and gelatin (18 – 21). Recently, we showed that N-Als1p has a lectin-like activity, since it interacts with fucose-containing carbohydrates (22). Despite the vast amount of information available on Als-mediated adhesion, there is still little data available on the Als molecular binding mechanisms mediated by host carbohydrates. Another prominent yeast adhesin family is the Epa (epithelial adhesin) family, since it has been reported to be mainly responsible for the adherence of *C. glabrata* to human cells (23–25). The N-terminal domains of Epa proteins (N-Epa-p) do not share sequence homology with the adhesins of the Als family, which are not present in *C. glabrata*. Rather, N-Epa proteins are classified as PA14-like lectins (26–28) because of their sequence homology and structural similarity to the PA14 fragment of the anthrax toxin protective antigen. They mediate adherence to human epithelial and endothelial cells by recognizing glycans containing terminal galactose residues (25) and show the highest affinity for the Thomsen-Friedenreich (T or TF) antigen (Galβ-1,3-GalNAc), which likely mediates N-Epa-p adherence to highly glycosylated proteins such as mucins (28). Although we recently demonstrated that wild-type N-Epa1p binds to FN from human plasma (29), no experimental data on the potential host glycoprotein binding receptors of Epa1p or other *C. glabrata* adhesins are available.

In viral host-pathogen interactions, lectin carbohydrate-binding agents (CBAs) can bind to viral envelope glycans and thereby inhibit the entry of, e.g., the human immunodeficiency virus (HIV) into host cells (30–33). A strong feature of lectin CBAs as potential antiviral drugs is their multifarious mechanism of action. They can inhibit viral replication and cell-cell transmission of viral particles and induce partial deletion of the envelope glycan shield, with consequent exposure of immunogenic epitopes to neutralizing antibodies. Moreover, these antiviral compounds do not need to be internalized by host cells to be effective against the virus (32). Various mannose-specific lectins endowed with potent antiretroviral activity have been discovered. They have been isolated from cyanobacteria, actinobacteria, algae, higher plants, and worms (34–36). Antiviral activity of lectin CBAs against viruses other than HIV with high-mannose glycosylated envelope proteins, such as influenza virus, herpesvirus, hepatitis C virus, dengue virus, Marburg virus (MARV), severe acute respiratory syndrome (SARS) coronavirus, measles virus, and Ebola virus, has been discovered (37–45).

In this report, we present a novel glycan array-based network strategy aimed at identifying the potential biological binding receptors for adhesin lectins. First, the glycan determinants of the lectins are determined from the experimentally evaluated glycan-binding specificities of the lectins by glycan array analysis. Next, the GlycoSuiteDB glycoproteomic database of the UniCarbKB platform (46, 47) is searched for these determinants to obtain a set of human glycoproteins expressing the glycan determinants that are of interest and are considered potential targets for lectin recognition. By performing additional queries of the GlycoSuiteDB database, these potential target glycoproteins can be further linked to the cell types on which they are present, the tissues and body systems, and the disease state (if applicable). Finally, the network is analyzed in order to profile the potential adhesin binding receptors in the host with prioritization, based on experimental binding data, of the most relevant interactions. New potential targets for the selected adhesins were predicted and experimentally confirmed. The constructed networks are referred to as lectin-glycan interaction (LGI) networks. We explored this strategy and constructed LGI networks for two classes of pathogenic microbial adhesins that are characterized by lectin-like properties, i.e., the bacterial UPEC adhesin FimH-LD, as well as the yeast *C. albicans* and *C. glabrata* adhesins (i.e., Als1p and Als3p and Epa1p, Epa6p, and Epa7p, respectively). The LGI networks constructed were corroborated by comparison with interaction data available in the literature, and some links in the networks were experimentally confirmed by quantitative lectin-glycan interaction analysis (by surface plasmon resonance [SPR] and atomic force microscopy–single-molecule force spectroscopy [AFM-SMFS]). This networking strategy was also used to predict lectin interactions with envelope glycoproteins of human-pathogenic viruses. It was successful in anticipating the molecular recognition of the HIV gp120 envelope protein by the bacterial adhesin FimH-LD, and it led to the discovery of the anti-HIV activity of FimH.

## RESULTS

### Modeling and visualization of LGI networks.

The proposed LGI network has been modeled as a weighted, undirected graph composed of a set of nodes (i.e., lectin, glycan, glycan determinant, glycoprotein, disease, tissue, body system) and a set of edges connecting the pair of nodes (see Materials and Methods and the supplemental material). An edge connecting the two nodes can represent biochemical interactions, as well as biological and/or hierarchical associations between the nodes. With each node, we associate a node relevance, and with each edge, we associate an edge relevance, which indicates the relevance of the interaction between the connected nodes. The proposed representation allows visualization of the network in multiple ways in order to (i) highlight a set of glycoproteins as promising receptor candidates that were obscured in the huge amount of data in the LGI network and (ii) predict the potential binding receptors for several lectins. In our specific case, the network involved a combination of both experimental data and the data from the publicly available GlycoSuiteDB database. The binding specificity and strength of microbial lectins (FimH-LD, N-Als, and N-Epa) were experimentally determined by glycan array screening. The measured binding strengths were exported into a spreadsheet, whereas those glycan structures and determinants that are recognized by the adhesins were queried in the GlycoSuiteDB database. The query provided a list of hundreds of potential target proteins (cases of glycan determinants not attached to any protein were also taken into account), and they were further linked to cell types, tissues, body systems, and diseases (if available) through additional queries in the same database. The resultant nodes and edges/links (associations) provided the LGI network definition in its initial state, where all of the nodes and edges were considered equally relevant; a relevance quantification stage followed.

**FIG 1:**
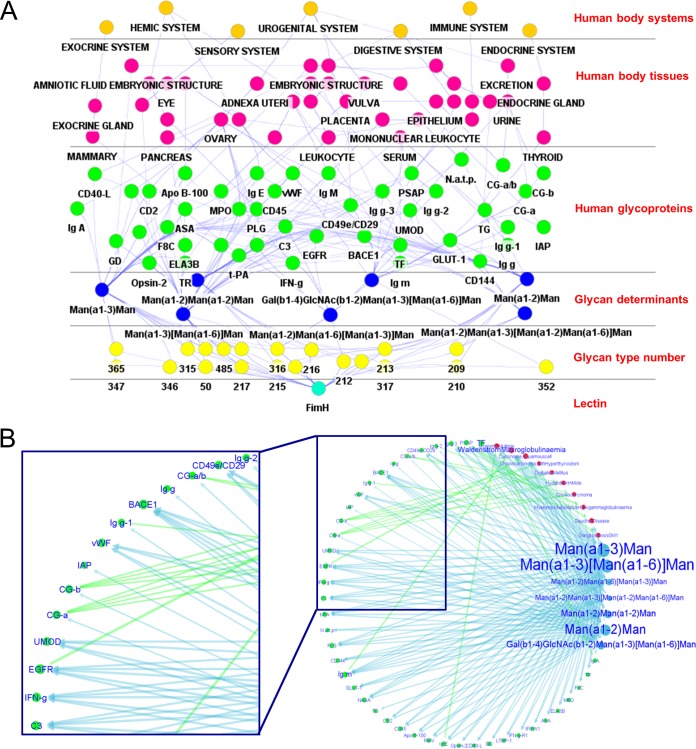
The FimH LGI network. (A) Hierarchical view of the FimH LGI network generated by using cytoscape with the cerebral app. The human proteins and the related abbreviations are listed in Table S1 in the supplemental material. (B) Circular view of the network (generated with the LGI network algorithm). The glycan determinants queried in the UniCarbKB database, the human glycoproteins bearing the glycan determinants, and the related diseases are depicted as blue, green, and red nodes, respectively. Connections between nodes are depicted with blue arrows (to indicate the determinants expressed on each glycoprotein) or green arrows (to indicate the diseases associated with the altered glycosylation of each glycoprotein). A closeup view of the network is shown on the left. The size of each node (and the font size of the node label) in the circular view is proportional to the number of connections with other nodes and the associated lectin binding intensity, which were experimentally determined by glycan array analysis; the arrow thickness is correlated to the lectin binding intensity.

**FIG 2:**
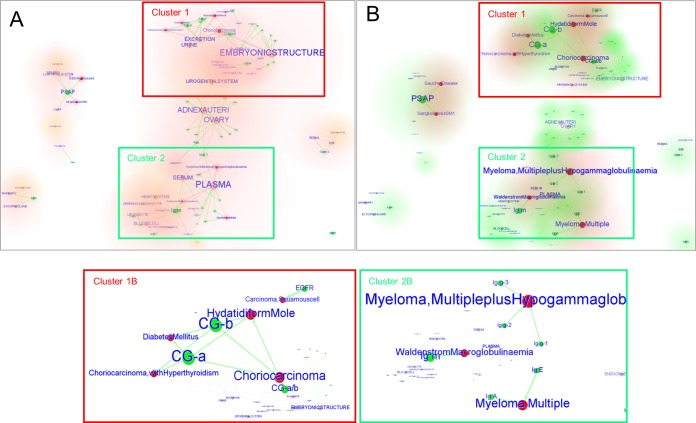
Cluster view of the FimH LGI network. The cluster representation shown is focused on the human glycoproteins (green nodes), tissues (pink nodes), systems (orange nodes), and diseases (red nodes) associated with glycan determinants (generated with the LGI network algorithm). The tissue clusters are depicted with a pink glow (A), the system clusters are depicted with an orange glow (A), the protein clusters are depicted with a green glow (B), and the disease clusters are depicted with a red glow (B). Two main clusters are present in the cluster view: cluster 1, containing glycoproteins and diseases linked to tissues of the urogenital system, and cluster 2, which is mostly associated with the hemic system. Closeup views of these clusters (B) are shown to indicate the most relevant nodes (lower panels). In panel A, the nodes related to the tissues are important, since they are the most connected ones (the larger the number of connections, the larger the node and font size of the label). In panel B, the disease and protein nodes are most important, since the node (and font) sizes are proportional to the lectin binding intensities, and they are the most connected ones. The thickness of the arrows is proportional to the associated lectin binding intensity.

**FIG 3:**
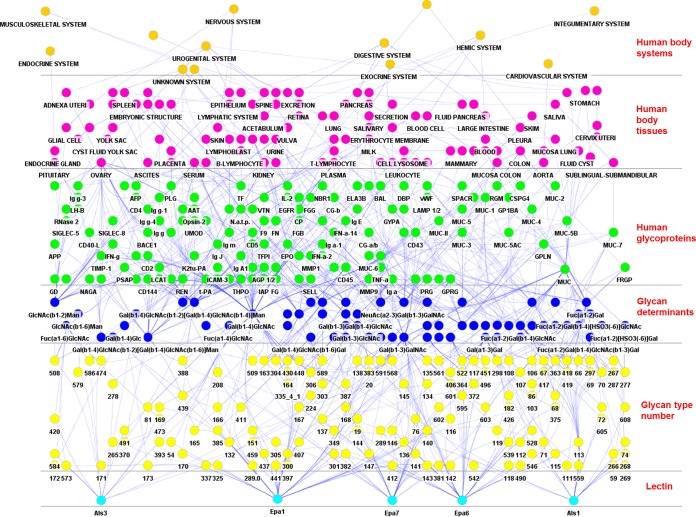
Hierarchical structure of the LGI network for the Als and Epa lectins. Hierarchical view from the lectin-like adhesins (bottom) toward the human body system (top). The human proteins and the related abbreviations are listed in Table S1 in the supplemental material.

**FIG 4:**
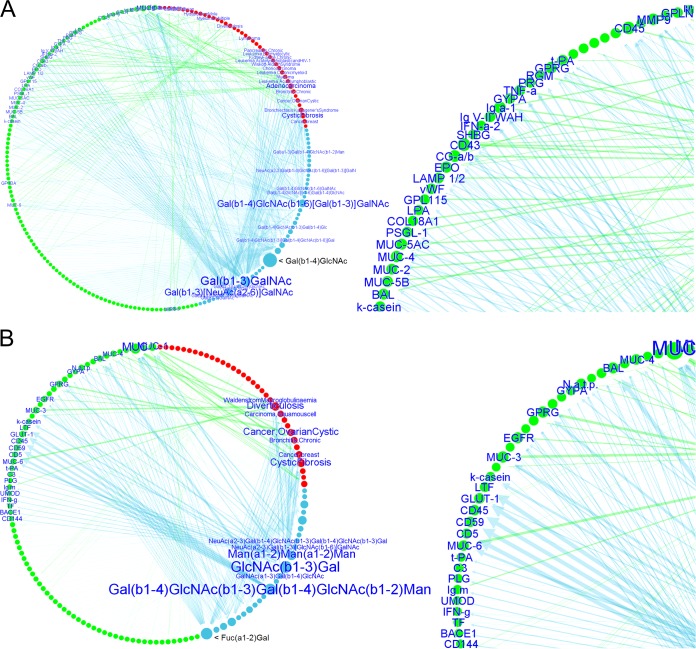
Circular views of the Epa-only and Als-only LGI network. Glycan determinant data and their connections with human glycoproteins and related diseases are depicted for *C. glabrata* N-Epa-p (A), and *C. albicans* N-Als-p (B). Closeup views of the networks are shown on the right. The nodes dimensions and arrow thickness/label size depend on the number of connections, and the glycan-binding strength, respectively (see the legend to Fig. 1). Notably, the determinants Gal(β1-4)GlcNAc (A) and Fuc(α1-2)Gal (B) are both characterized by a high number of connections (large node, i.e., several human glycoproteins are characterized by the presence of these glycan determinant) but a low relevance. No label is shown; i.e., the Epa/Als intensities of binding to the glycans that contains these determinants are lower than the other determinants. The program filters out the node labels on the basis of the lectin binding intensity and displays only the labels of the most relevant glycan determinants (see the supplemental material).

**FIG 5:**
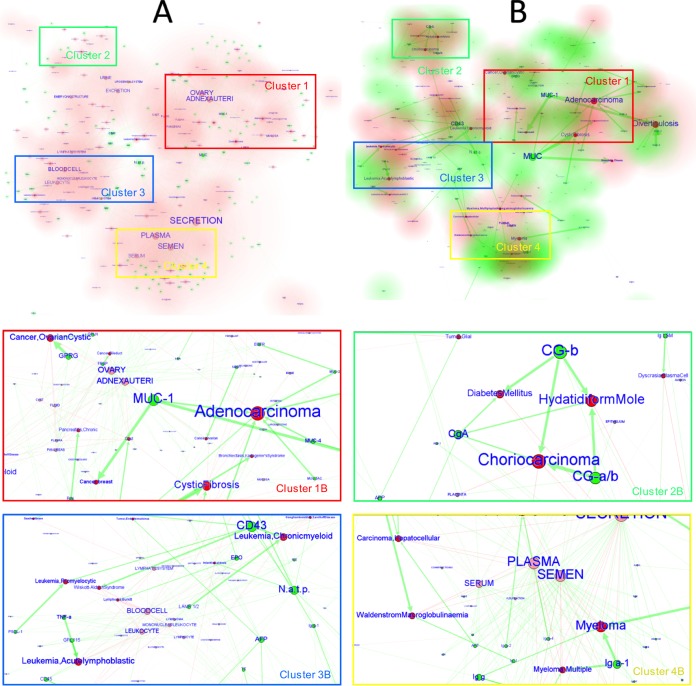
Cluster views of the global Als/Epa LGI network. This representation is focused on the human glycoproteins, tissues, systems, and diseases associated with the glycan determinants recognized by the Als/Epa adhesins. Tissue and disease clusters are highlighted in panels A and B, respectively. The tissue clusters are represented with a pink glow (A), the protein clusters are shown with a green glow (B), and the disease clusters are shown with a red glow (B). The most relevant elements of the four main network clusters are shown in detail as closeup views of panel B (four lower subpanels). In panel A, the nodes related to the tissues are important, since they are the most connected ones (the larger the number of connections, the larger the node and font size). In panel B, the disease and protein nodes are most important, since the node (and font) sizes are proportional to the lectin binding intensities, and they are the most connected ones. The thickness of the arrows is proportional to the associated lectin binding intensity.

The relevance quantification process was achieved in two steps. We first associated each edge with a relevance depending on the type of its connecting nodes (i.e., protein-glycan) according to the proposed weights (see equations 1 to 3 in the supplemental material) as a function of the lectin binding intensity measured via glycan array screenings. In the second step, the node’s relevance was estimated by using a network analysis centrality measure (48) that involves both the number of edges (associations) connected to the node and their weights (lectin binding intensity) (see the supplemental material). The influence of the number of edges (or associations) versus the binding intensity is regulated by the parameter λ (see the supplemental material for a discussion of its influence), and its value can be interactively adjusted during the visualizations to better study the different associations (see Fig. 2 and 5). Determination of the relevance values allowed us to rank or establish priorities among the potential targets, enabling a convenient visualization of the most important proteins (or disease, glycan, etc.) as the bigger nodes and the most important associations (i.e., protein-disease, disease-glycan) as thicker edges/links. In particular, three complementary visualization types were used, namely, hierarchical, circular, and cluster views (11). This allowed an interactive analysis of the resultant network in great detail, highlighting the most relevant nodes in the overall network, as well as within specific clusters, at the same time (see the supplemental material). The hierarchical view (see Fig. 1, 3, and 7) allowed linking of the experimentally determined lectin specificities (i.e., the glycan determinants) with the potential receptors (human or viral glycoproteins) and then linking of these glycoproteins to cell types/tissues and body systems. The circular view allowed easy identification of all of the relevant nodes according to their size, and the most important associations were made as the thickest arrows (see Fig. 1 and 4). The cluster view revealed cluster-like visualizations (49), thus allowing us to explore groups of nodes belonging to the same local cluster (see Fig. 2 and 5). Some glow and shading effects can be (optionally) applied in order to distinguish between the groups of nodes according to their types within the same cluster (see Fig. 2A and B and 5A and B).

The results of network analysis both exhibited known associations and predicted novel ones, proposing novel candidates (i.e., glycoproteins and glycan determinants) as promising targets for later adhesin binding experiments. Notably, the same network analysis can be performed to predict the binding receptors of any carbohydrate-binding protein in other host organisms once the experimental carbohydrate-binding characterization of the lectin has provided the molecular binding strength and the association data.

### The LGI network of *E. coli* FimH-LD with predicted human receptors.

The FimH LGI network (Fig. 1) was generated by using FimH-LD experimental glycan array data that are perfectly consistent with the previously published microarray results (10) and reflect the high specificity of FimH-LD for high-mannose glycans, i.e., the oligomannose 3, oligomannose 5, mannotriose, and mannopentaose structures. A hierarchical view of the network shows the predicted receptors (human glycoproteins) for the FimH adhesin and their classification in terms of body tissues and systems (Fig. 1A). A circular view of the same LGI network (Fig. 1B) focuses on the glycan determinants that are bound by FimH-LD, the potential glycoprotein ligands, and also lists the diseases correlated with the expression of the glycosylation determinants indicated. Only a few of the predicted glycoprotein binding receptors are associated with specific disease states (mainly malignancies), which is the case for several immunoglobulins (such as, IgE, IgM, and IgA), CG-a, CG-b, the epidermal growth factor receptor (EGFR), and PSAP (see Table S2 in the supplemental material for a list with abbreviated protein names). Among these, immunoglobulin mu (IgM) and the EGFR are reported by the network to be particularly relevant, while TF is highlighted in the group of non-disease-associated glycoproteins. The latter are all linked to the determinants containing either Manα-1,2-Man or Manα-1,3-Man as terminal moieties. The network displays other human glycoproteins, such as UMOD, CD49e/CD29, C3, t-PA, and PLG, for which a link with FimH or type 1 fimbriae has already been established (see Discussion) and for which any defined relationship with the bacterial adhesin has not been found yet, for example, BACE1, vWF, and gamma interferon.

The cluster view of the network shows the identification of the host tissues that could be potentially targeted by the adhesin and the disease states that could promote the binding of the adhesin to the predicted glycoprotein receptors (Fig. 2). Two main clusters can be identified in the cluster views of the network. Cluster 1 contains glycoproteins and diseases linked to the tissues of the urogenital system, and cluster 2 is associated with the hemic system. In cluster 1 are proteins such as UMOD, CD49e/CD29, CG-a, and CG-b, which are found in several tissues of the urogenital tract and constitute a relevant subcluster. The latter two glycoproteins (CG-a, CG-b) are particularly highlighted in the second view of the network (Fig. 2B) and are associated with choriocarcinoma, hydatidiform moles, and diabetes mellitus (Fig. 2, cluster 1B). The same cluster also contains glycoproteins that are present in a healthy uterus and ovary. Cluster 2 is mainly characterized by the presence of links between immunoglobulins, namely, IgA, IgM, IgE, and IgG1/2/3, and different forms of blood cell cancer, such as myelomas and Waldenström’s disease (Fig. 2, cluster 2B).

### The LGI network of *Candida* N-Als-p and N-Epa-p with predicted human receptors. (i) Modeling and visualization of the LGI network of N-Als and N-Epa lectins from *Candida*.

The hierarchical view of the overall Epa/Als network (Fig. 3) is extended, since it is based on the glycan specificities of six adhesins. Mucins (several proteins containing the abbreviation MUC) appear as the most relevant human glycoproteins. In the circular view of the Epa network (Fig. 4A), mucins are mainly connected to the glycan determinants Galβ-1,3-GalNAc, Galβ-1,4-GlcNAc, and Galβ-1,4-GlcNAcβ-1,6[Galβ-1,3-]GalNAc. These three determinants, together with the sialyl-T antigen, are prioritized on the basis of the related lectin binding intensities, especially in the case of the Galβ-1,3-containing moieties (large arrow width), and the number of connections, i.e., for Galβ-1,4-GlcNAc, the largest node size in the network but unlabeled because of the low binding intensity (the label was filtered out in the program for better visualization of the more relevant nodes [see the supplemental material]). In the Als network (Fig. 4B), mucins are mainly linked to the GlcNAcβ-1,3-Gal determinant, which is also the most relevant (high binding intensity) in this network. Other interesting links with glycoproteins that have already been discussed in the literature (k-casein, EGFR, CD144, LTF, TF, IgM) or need to be confirmed (tumor necrosis factor alpha, PSGL-1, CD43, CD45, vWF, BACE1, LAMP1/2, PLG) emerge from the two networks (Fig. 3) (see Discussion). Among the Als glycan determinants, the di-LacNAc determinant (Galβ-1,4-GlcNAcβ-1,3-Galβ-1,4-GlcNAcβ-1,2-Man) emerges and is based on experimental high binding intensity (Fig. 4B), while the Fucα-1,2-bearing structures, especially Fucα-1,2-Gal (largest unlabeled node), are relevant because of their large number of connections. Several disease nodes are present in both networks, such as cystic fibrosis, diverticulosis, and different forms of cancer, while adenocarcinoma is highlighted in the Epa network and is associated mainly with mucins (Fig. 4A).

Four main clusters are identified in the Epa/Als network (Fig. 5). The most relevant links are found in cluster 1, and it involves mucins; the diseases associated include lung and intestinal adenocarcinomas (MUC, MUC1, MUC2, MUC4, MUC5AC/B), diverticulosis (MUC1, MUC3, MUC4, MUC6), cystic fibrosis (MUC, MUC7), chronic bronchitis, breast cancer (MUC1), and Kartagener’s syndrome (bronchiectasis) (MUC). In cluster 2, the glycoproteins associated with the urogenital system, such as choriogonadotropin (CG), and some related malignant states, such as choriocarcinoma and diabetes mellitus, are identified. In cluster 3, glycoproteins CD43 and CD45 are strongly associated with Wiskott-Aldrich syndrome (WAS) (CD43), together with different forms of leukemia (CD43/CD45) affecting cells of the immune system and also coexisting with HIV infections (CD45). Another important link is established by glycan determinants, which are not attached to any protein (mainly fucosyl-capped oligosaccharides for Als adhesins and nonreducing terminal Gal-GlcNAc moieties for Epa adhesins). They are connected to chronic kidney failure, gangliosidosis, fucosidosis, and sialidosis. Additionally, associations were found in cluster 4, mainly linking myelomas with several immunoglobulins and other plasmatic glycoproteins and glycoproteins of the secretion system.

### (ii) Validation of Als/Epa predicted interactions: binding of N-Als3p to FN and laminin and of N-Epa1p to mucin.

**FIG 6:**
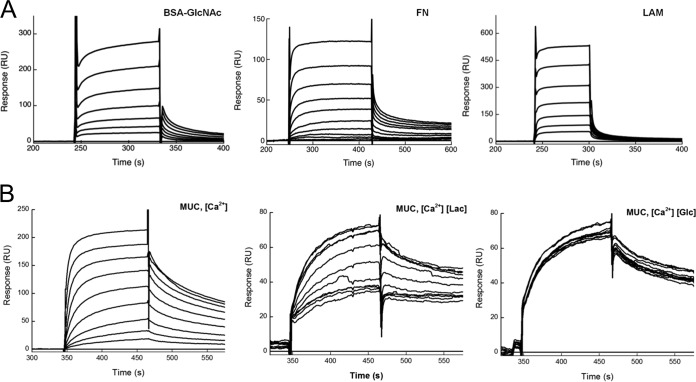
Binding characterization of N-Als3p and N-Epa1p. (A) SPR sensorgrams of N-Als3p binding to BSA-GlcNAc (1.5 to 99 µM) (left), FN (0.16 to 40 µM) (center), and laminin (LAM) (2.6 to 170 µM) (right). (B) SPR sensorgrams of N-Epa1p binding to mucin (MUC) (100 µM to 39 nM) (left), and binding inhibition experiments with increasing concentrations (0 and 6 µM to 1.5 mM) of lactose (Lac, center) and glucose (Glc, right). RU, relative units.

Glycan array screening was performed to determine the glycan specificities and affinities of N-Als3p (see Fig. S2A to D in the supplemental material). SPR experiments confirmed the binding of N-Als3p to GlcNAc (Fig. 6A), as well as the binding of N-Als3p to FN and laminin (Fig. 6A), two proteins of the extracellular matrix (ECM) also recognized by N-Als1p (22). The N-Als3p dissociation constant at the equilibrium state (*K_D_*) for the bovine serum albumin (BSA)-GlcNAc glycoconjugate was estimated in the micromolar range (*K_D_* = 34 ± 4 µM). The *K_D_* constants were 10 ± 1 µM for the N-Als3p–FN interaction and 410 ± 40 µM for the N-Als3p–laminin interaction. The affinity of N-Epa1p for mucin was also determined by SPR (Fig. 6B), and the *K_D_* constant was 4.67 ± 0.87 µM. In order to verify that the observed interactions were specifically mediated by galactose-containing glycans attached to FN and mucin, binding inhibition experiments were performed (Fig. 6B). The binding of N-Epa1p to mucin could be blocked by lactose in a concentration-dependent manner, but it was not affected by the presence of glucose.

### The LGI network of *E. coli* FimH-LD with viral envelope glycoproteins. (i) Modeling and visualization of the FimH-LD LGI network with viral envelope glycoproteins.

**FIG 7:**
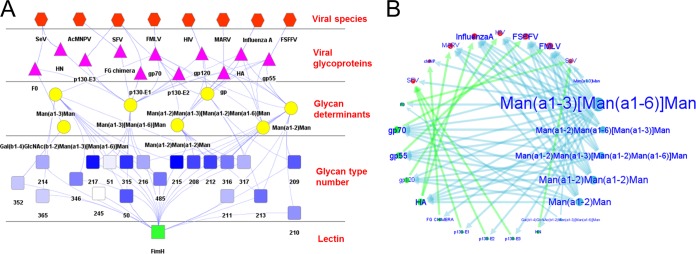
The FimH-LD–viral LGI network. (A) Hierarchical view of the FimH-viral LGI network from the lectin (bottom) toward the viral species (top). Abbreviations: MHV, murine hepatitis virus; SeV, Sendai virus; SFV, Semliki Forest virus; AcMNPV, Autographa californica multiple nucleopolyhedrovirus; FMLV, Friend murine leukemia virus; FSFFV, Friend spleen focus-forming virus. (B) Circular view of the network including the glycan epitopes, the viral glycoproteins, and the viral species data. The size of each node (and the font size of the node label) in the circular view is proportional to the number of connections to other nodes and the associated lectin binding intensity; arrow thickness is correlated with lectin binding intensity.

The FimH-viral network displays the connections of FimH-LD and the glycans (recognized by the lectin on the glycan array) to several viral glycoproteins through glycan determinants (Fig. 7A). Connected viruses include HIV, Sendai virus, Friend murine virus, MARV, and influenza A virus. The gp120 envelope glycoprotein of HIV is included in the network, and this interaction was explored further (see below). Interestingly, FimH-LD is also linked to influenza A virus hemagglutinin (HA) (from insect cells and chicken isolates) and to the MARV envelope glycoprotein (gp; from monkey isolates). This may anticipate the possible interactions between the LD of FimH and the envelope of these critical viral pathogens. The circular view (Fig. 7B) predicts the most relevant adhesion epitopes for FimH on viral glycoproteins, such as influenza A virus HA and MARV gp, that are connected to several high-affinity glycan epitopes (especially to Manα-1,3-[Manα-1,6-]Man).

### (ii) Validation of *E. coli* FimH-LD predicted interactions with viral envelope glycoproteins.

**FIG 8:**
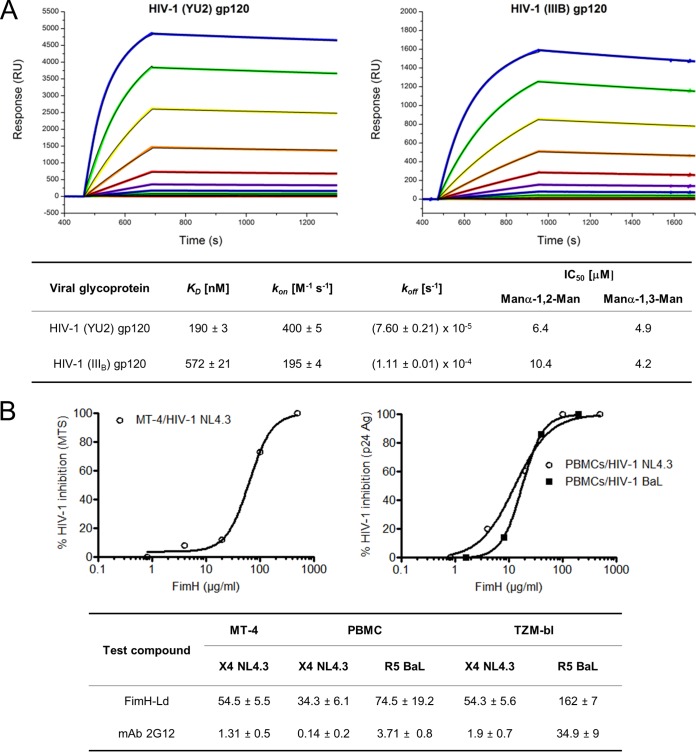
Characterization of FimH-LD and N-Epa1p interaction and FimH-LD anti-HIV activity. (A) SPR sensorgrams of FimH-LD binding to HIV-1(YU2) gp120 and HIV-1(III_B_) gp120. The experimental data (rainbow-colored curves) were fitted with a one-binding-site model (black curves). The dissociation constants, kinetic parameters, and inhibition constants of FimH-LD–gp120 interactions are shown at the bottom. RU, relative units. (B) Inhibition graphs and related EC_50_s of FimH-LD and anti-gp120 MAb 2G12 (μg/ml) for the different antiviral assays.

The affinity of the LD of FimH for gp120 from HIV-1(III_B_) and HIV-1(YU2) was kinetically characterized by SPR (Fig. 8A). FimH-LD shows very low association rate constants (*k_on_* values 2 to 4 M^−1^ ⋅ s^−1^), but also very low dissociation rate constants (*k_off_* values ≤10^−4^ s^−1^). The difference between the calculated *K_D_* parameters for the two interaction couples is related mainly to the 2-fold higher *k_on_* in their binding with baculovirus-derived gp120. This discrepancy can be explained after considering that protein glycosylation processes are different in baculovirus and CHO expression systems.

To confirm that the recognition of gp120 by FimH-LD is mediated by the glycan moieties on the viral protein, SPR inhibition experiments were performed with Manα-1,2-Man and Manα-1,3-Man, which mimicked different moieties of the viral protein glycosylation sites. Fifty percent inhibitory concentrations (IC_50_s) were calculated by using the response values at equilibrium (*R_eq_*) at a constant FimH-LD concentration (Fig. 8A). The interaction of FimH-LD with the viral envelope protein could be blocked in a dose-dependent manner by both disaccharides that exhibited a similar inhibitory concentration but a slightly higher specificity for the α-1,3-linked mannobiose.

An additional confirmation of the interaction of FimH-LD with gp120 was obtained by AFM-SMFS. Adhesion force histograms resulting from FimH-LD–gp120 (YU2) unbinding events showed a broad range of interactions occurring at forces that are relatively high for a noncovalent bond (see Fig. S1 in the supplemental material). Unbinding force distributions with an average peak force of 225 ± 18 pN (see Fig. S1C) were obtained. This interaction force value is comparable with that observed for fimbrial tip adhesion to BSA-mannose (50). The FimH-LD–gp120 interactions could be blocked almost completely in the presence of α-1,3-mannobiose (see Fig. S1B).

FimH-LD anti-HIV activity was assessed in different *in vitro* cell assays (Fig. 8B). The lectin activity (50% effective concentration [EC_50_]) against X4 HIV-1 NL4.3 was 54.5 µg/ml in the MT-4 cell line, as evaluated by the induction of a CPE and subsequently by the 3-(4,5-dimethylthiazol-2-yl)-5-(3-carboxymethoxyphenyl)-2-(4-sulfophenyl)-2H-tetrazolium (MTS)–phenazine ethosulfate (PES) method. In addition, when the protein was evaluated against X4 HIV-1 NL4.3 and R5 HIV-1 BaL replication (measured by P-24 HIV-1 antigen [Ag] enzyme-linked immunosorbent assay [ELISA]) in peripheral blood mononuclear cells (PBMC; obtained from healthy donors), the EC_50_s obtained were 34.3 ± 6.1 (*n* = 8) and 74.5 ± 19.2 (*n* = 6) µg/ml, respectively. These anti-HIV-1 data show a consistent anti-HIV-1 activity profile, although the peptide was somewhat less active (~2-fold) against R5 HIV-1 replication. The 50% cytotoxic concentration was >500 µg/ml, and there was no cytotoxicity observed at all at 500 µg/ml in MT-4 cells and in PBMC, which provided a selectivity index of at least >10 for X4 HIV-1. As a control, broadly neutralizing anti-gp120 carbohydrate monoclonal antibody (MAb) 2G12 was included in these experiments. When evaluated in the same PBMC HIV replication assay in our experiments, it showed EC_50_s of 0.14 µg/ml for HIV-1 NL4.3 and 3.71 µg/ml for HIV-1 BaL. So, the slight decrease in anti-HIV activity of FimH-LD against R5 HIV-1 BaL is very often observed with other CBAs and other classes of compounds. The antiviral activity of FimH-LD was also assessed with the TZM-bl–HIV-1 luminescence assay. The infectivity of the TZM-bl cell line with X4 HIV-1 NL4.3 was inhibited with an IC_50_ of 54.3 µg/ml, while the inhibition of R5 HIV-1 BaL infectivity generated an EC_50_ of 162 µg/ml. Again, MAb 2G12 was used as the control when EC_50_s of 1.9 µg/ml for X4 HIV-1 NL4.3 and 34.9 µg/ml for R5 HIV-1 BaL were obtained.

## DISCUSSION

### The LGI network of the *E. coli* FimH-LD adhesin.

The FimH LGI network strategy developed pointed out a limited number of human glycoproteins, mainly situated in the urogenital and hemic systems of the human body, as the potential binding receptors for the FimH adhesin. Only a few of the predicted links have been experimentally confirmed in the literature, and these deliver the first proof of the validity of our strategy for the prediction of lectin binding receptors.

Type 1 fimbriated *E. coli* is able to recognize Tamm-Horsfall glycoprotein, also known as uromodulin (UMOD) (51). The binding to this protein was confirmed by the network strategy (Fig. 1). UMOD is a urinary defense factor, since it can prevent the interaction of bacterial fimbriae with the uroplakin receptors through its single glycan chain. FimH is also involved in the invasion of uroepithelial cells because of its direct binding to high-mannose glycans of integrins β1 and α3 (52). Other integrin subunits could also be involved in the entry of FimH-expressing bacteria into host cells (52). One of these subunits could be the α5 integrin, which was also predicted in the network, together with the β1 integrin as a α5/β1 heterodimer (CD49e/CD29) (Fig. 1) that was already reported to mediate host cell invasion through the pathogenic bacterium *Staphylococcus aureus* (53). Type 1 fimbriae also have a role in complement-dependent bacterial internalization (54). Synergy between the expression of FimH on bacterial fimbriae and binding of the C3 complement protein increased *E. coli* internalization through epithelial cells of the urinary tract. This effect was abrogated by mannose or in the absence of the mannose-specific adhesin on the bacterial fimbriae. The same synergistic effect could not be correlated with the expression of P fimbriae, which would be carrying the PapG adhesin. These data and the presence of C3 in the FimH LGI network suggest that the glycosylated complement protein, scavenged by and directly associated with the FimH LDs, could facilitate bacterial entry into uroepithelial host cells. A genetically engineered *Pseudomonas aeruginosa* strain that expresses type 1 fimbriae can specifically adhere to breast cancer cells overexpressing the EGFR and block the EGFR signaling pathway (55). The effect of fimbriae on receptor signaling has been described as mannose sensitive. Indeed, the LGI network suggests the recognition by FimH-LD of the EGFR from carcinoma cells. We can thus hypothesize that FimH-LD may also mediate bacterial adhesion and have the same effect on growth factor receptor signaling. The FimH-LD LGI network displays several immunoglobulin families that are associated with different malignant diseases, including IgA from myeloma cells (Fig. 1). Immunoglobulin preparations containing secretory IgA or IgA from myeloma cells can induce mannose-dependent agglutination of type 1 fimbria-expressing *E. coli* (56). Weaker agglutination of these cells can also be achieved through certain IgM isotypes and prevented by the presence of d-mannose. Other connections are established in the network between the fimbrial adhesin and disease states affecting protein glycosylation. For example, a link with diabetes mellitus is corroborated by the observation that female patients with this disease are more susceptible to UTIs than are healthy patients because of the greater adherence of type 1 fimbriated *E. coli* to bladder cells (57).

### The LGI network of *Candida* Als and Epa adhesins 

N-Als1p has been shown to interact with fucose-containing glycans that are present in blood group antigens and preferentially with antigen H type 2 (22). Therefore, we performed glycan array screening to also determine the carbohydrate-binding specificity of N-Als3p (see Fig. S2A to D in the supplemental material). Among the strongest binders, we found long chains of repeated LacNAc (Galβ-1,4-GlcNAc), and a micromolar affinity was determined for the interaction of the adhesin with BSA-GlcNAc. GlcNAc constitutes of a part of the type 1 LacNAc (Galβ-1,3-GlcNAc) and type 2 LacNAc (Galβ-1,4-GlcNAc) structures that build the scaffold for blood group H and Lewis-type units (58). Some human pathogens use the GlcNAc residue as a binding receptor; e.g., the fimbrial adhesin F17-G of enterotoxigenic *E. coli* binds to *N*-acetylglucosamine-presenting receptors on the microvilli of the intestinal epithelium of ruminants (59). Our N-Als3p glycan array data, together with data related to N-Als1p and the Epa adhesins, were used for the generation of LGI networks (Fig. 3 to 5). These networks revealed that a large set of potential binding receptors that may be recognized by these adhesins through their interaction with carbohydrates are displayed on human cells or present in body fluids. The most interesting ones are summarized in Table S1 in the supplemental material. Several glycan determinants are linked to mucins (Fig. 3 and 4). Mucins are the main constituents of the extracellular secreted mucus and cell surface glycocalyx, which is rich in the GalNAc-containing structures commonly used by many pathogens for adhesion. *C. albicans* adhesion to human cells has been previously linked to mucins (60, 61). The binding of Epa adhesins to mucin-type O-glycans has also been described, especially the ability of Epa1p, Epa6p, and Epa7p to recognize the T antigen (25, 27, 28). This disaccharide constitutes of the core 1 structure of mucin-type O-glycans, and it is mainly exposed on the surface of colon cancer tissues in a truncated form. The T and sialyl-T antigens are also found on breast cancer cells (62). The three Epa proteins are linked in the network to the mucins found in several tissues or fluids, such as saliva, lung tissue, stomach tissue, mammary gland tissue, milk, colon tissue, and uterine tissue. A consistent fraction of these mucins is associated with diseased states, i.e., colon adenocarcinoma (MUC1, MUC2, MUC4, MUC5A/B/C), breast and uterine cancers (MUC1), and lung diseases, which may cause bronchiectasis (MUC) (Fig. 5). These mucins carry the T antigen, the sialyl-T antigen, or both. A link between Epa adhesins and the colon mucosa has been demonstrated by showing adhesion of *Saccharomyces cerevisiae* cells expressing Epa1p and Epa6p to human colorectal carcinoma (Caco-2) cells (28). A connection between the Epa proteins and CD43 (leukosialin)/CD45 (receptor-type tyrosine-protein phosphatase C) is also present in the LGI network (Fig. 4), and this is justified by the presence of the T antigen that is linked to leukemia- and HIV-associated CD45 and to WAS-associated CD43, as well as other glycans related to a healthy condition. CD43 and CD45 receptors are commonly expressed on leukocytes (63). They are involved in lymphocyte activation and may present altered glycosylation in HIV-infected cells (64–66) or in diseases such as leukemia and WAS. Lysosome-associated membrane glycoproteins 1 and 2 (LAMP1, LAMP2), which are as well predicted, are expressed in macrophages and are essential for the fusion of phagosomes and lysosomes during phagocytosis (67). They may also present altered glycosylation in leukemia cells. Epa1p is able to mediate yeast adhesion to human leukemic macrophages and healthy PBMCs, in order to trigger cytokine expression and induce phagocytosis (68). The inhibitory effect of phagocytosed *C. glabrata* on the fusion between phagosomes and lysosomes in macrophages is an efficient immune evasion strategy (69). Possibly, Epa1p adhesion to human PBMCs is mediated by CD43/45. This is consistent with CD43/45-mediated adherence of *Actinomyces naeslundii*, expressing Galβ-1,3-Gal(NAc)-specific adhesins, to polymorphonuclear leukocytes or promyelocytic leukemia cells (70) and the involvement of CD45 in the induction of cytokine production, as for the T-antigen-specific jacalin (plant lectin) that recognizes CD45 on T lymphocytes and induces cytokine secretion (71).

Epa proteins are linked to FN (Fig. 3); i.e., Epa1p and Epa7p are linked to FN of fibroblasts by LacNAc-terminated N-glycan branches, suggesting again the specificity of only these two Epa adhesins for the ECM protein. The link of N-Epa1p with FN is corroborated by our recent results (29), which show that the adhesin domain is able to bind FN with submicromolar affinity in a carbohydrate-sensitive manner. On the other hand, a connection between Als proteins and FN was not found in the network, although we demonstrated here and previously that N-Als1p and N-Als3p both recognize FN (22).

The network shows that Als and Epa adhesins may bind to ceruloplasmin (CP), (sero)transferrin (TF), and lactotransferrin (or lactoferrin [LTF]) (Fig. 3 and 4). The first two are blood plasma glycoproteins, while the latter is present in different exocrine secretions. All of them are involved in iron metabolism. *C. albicans* is able to acquire iron from TF (72). Thus, *C. albicans* iron acquisition from host TF may be Als mediated, as is iron acquisition from human ferritin (73). Inhibitory effects of TF, IgM, and another components of Cohn fraction IV (most probably ceruloplasmin) on the growth of *C. glabrata* have been discussed (74), as has the capacity of a special LTF formulation to impair yeast adherence to vaginal epithelial cells (75). The EGFR and cadherin-5 (CD144) are also indicated in the network as potential ligands of both Als proteins (Fig. 4). For the first glycoprotein, there is agreement with a study describing the EGFR and HER2 as interaction receptors for Als3p (76). The interaction of Als3p with these receptors triggers their autophosphorylation, which leads to endocytosis of *C. albicans* by host cells. It has also been demonstrated previously that Als3p binds to N- and E- cadherins, which are present on endothelial and epithelial cells, respectively (77). As shown in Table S1 in the supplemental material, additional reported experimental results confirmed some Als LGI network nodes, such as for chondroitin sulfate proteoglycan 4, κ-casein, and CG-a/b.

### FN, laminin, and mucin recognition by *Candida* adhesins.

Using SPR, we characterized the interaction of N-Epa1p with mucin (Fig. 6B). A comparable *K_D_* value was found for the N-Epa1p–FN interaction (29). The specific binding inhibition by lactose corroborated the specificity of glycan recognition by N-Epa1p. These data confirmed, for the first time, the ability of N-Epa1p to bind mucins. As shown in the LGI network, adhesion of Epa adhesins to mucins is extremely relevant in the context of host adherence and is mediated by multiple O-glycan determinants.

The binding of N-Als3p to ECM glycoproteins, such as FN and laminin, was also characterized by SPR (Fig. 6A). Compared to N-Als1p–FN binding (22) and the N-Epa1p–FN interaction (29), the N-Als3p–FN interaction was >5- and 10-fold weaker, respectively. Also, the N-Als3p–laminin dissociation constant was higher than that previously determined for N-Als1p. The full-length Als1 and Als3 adhesins showed significant binding to FN and laminin (18). It seems that the interaction of Als proteins with FN is determined mainly by the protein-protein interactions. Indeed, fucose failed to inhibit N-Als1p binding to the glycoprotein, while glucose and galactose only partially inhibited it (22). Additionally, we could not find a connection between Als adhesins and FN in the LGI network. Recently, it was shown that NT-Als9-2p recognizes the C-terminal sequence of the fibrinogen γ peptide (78). These results indicate that protein-protein interactions may dominate the binding of N-Alsp to FN.

### The LGI network of the *E. coli* FimH-LD adhesin with viral envelope proteins and the anti-HIV activity of FimH-LD.

Several viral pathogens—such as HIV, influenza virus, SARS virus, hepatitis C virus, MARV, and Ebola virus—contain high-mannose glycans attached to the envelope proteins (40). To predict interactions of FimH-LD with viral envelope glycoproteins, we generated a viral LGI network by employing FimH-LD glycan array data and the viral glycoproteomic data available in the GlycosuiteDB database. The network suggested the recognition of different viruses, including three human-pathogenic viruses (Fig. 7). One of these predictions, i.e., the link with HIV, was experimentally and thoroughly validated by using biomolecular and cellular assays.

The gp120–FimH-LD SPR affinity and inhibition results are compatible with a prevalent recognition of oligomannose 9 glycans on CHO-derived gp120. The related *K_D_* (572 nM) is, indeed, very similar to the affinity of the LD for the same carbohydrate structure (~400 nM) (11). By interacting with this longer oligosaccharide, the lectin would recognize the terminal α-1,2-linked mannose units rather than the Man-α-1,3-Man-β-1,4-GlcNAc, which is only recognized in a terminal configuration. On the other hand, we can explain the higher affinity for baculovirus-derived gp120 by hypothesizing the binding to a mixture of “long” and “short” glycans, including oligomannose 3/5. The IC_50_s reflect the actual preference of FimH-LD for α-1,3-linked mannosides over the α-1,2-linked oligosaccharides.

Envelope glycans are becoming increasingly promising targets for lectins as viral entry inhibitor proteins (32, 33). The kinetic analysis results of FimH-LD–gp120 interactions revealed association constants 2 to 4 orders of magnitude lower than those for other antiviral lectins, such as cyanovirin (CVN), actinohivin, and griffithsin (GRFT), but also, the dissociation rates are slower than those for the other antiviral lectins (79, 80). Accordingly, the resulting equilibrium constants for the same interactions were much higher than the *K_D_* of the best-characterized lectins CVN and GRFT, but they are still in the nanomolar range. This affinity of FimH-LD for the viral envelope protein justifies the moderate *in vitro* antiviral activity of the lectin. Comparable anti-HIV activity values were obtained for FimH-LD, and this in cellular assays using CD4^+^ T lymphocytes, TZM-bl cells, and PBMCs against CXCR4-using (X4) and CCR5-using (R5) HIV-1 strains (Fig. 8B). Especially the PBMC data are very relevant, since these are the real target CD4^+^ T cells for HIV.

Envelope glycoproteins from MARV and influenza A virus are also connected in the network as potential targets for FimH-LD (Fig. 7A). The mannose-binding lectin, a protein belonging to the innate immune system and specific for mannose-containing glycans, is able to hamper both influenza A virus (81, 82) and MARV (83) infectivity and spreading *in vitro*, which represents the body’s first line of defense against infection. Moreover, CVN activity against Ebola virus, MARV (39), and influenza A virus H1N1 isolates (84) and antiviral properties of mannose-specific lectins from algae against influenza A virus (43) have been demonstrated. On this basis, it would be worth further exploring the antiviral properties of FimH-LD not only against HIV but also against other viral species, especially influenza A virus and MARV, in order to evaluate the possibility of the development of a multivalent drug that would be effective in the prophylaxis for different pathogens.

### Conclusions.

We developed and successfully employed a novel LGI network in which the carbohydrate-binding properties of the *E. coli* adhesin FimH and *Candida* adhesins from the Epa and Als adhesin families were explored. This LGI network strategy, based on glycan array screening results and a glycoprotein database inquiry, allowed the profiling of potential glycoprotein binding targets for the selected adhesins. We confirmed some of these potential targets either experimentally or by reference to the literature. Additionally, this strategy was adapted for the prediction of adhesin-viral glycoprotein interactions, validated by the discovery of anti-HIV activity of FimH. This study shows the potential of the new strategy for the study of some microbial and viral interactions.

The LGI networks presented were based on the glycan array results coming from the database of the Consortium for Functional Glycomics (CFG). Experimental data could also be extracted from other databases (such as the Glycosciences Laboratory Database, Imperial College London). Care should be taken to check the quality of the extracted data. Although the Glycosuite database of UniCarbKB used is a curated glycoproteomic database, it has some limitations, since it is not exhaustive. We expect future improvements in the content of this database and, consequently, an enhancement of the LGI network’s prediction quality and accuracy. The LGI network strategy could be connected to additional bioinformatic resources that could support the validation of the LGI network generated, for example, the SugarBind database (http://sugarbind.expasy.org), which provides information on known carbohydrate sequences to which pathogenic organisms or substances (bacteria, toxins, and viruses) specifically adhere to. The network strategy developed could also be easily extended to other lectin-glycan interactions, where—besides human or viral interactions—other mammalian, plant, or protozoan glycan interactions could be explored, and also used, for example, for the discovery of new antimicrobial agents. Furthermore, the strategy could be used to predict the binding of viral proteins to human glycoproteins or carbohydrate structures, with the aim to facilitate the design of novel antiviral drug compounds, especially in the case of emerging viral pathogens.

## MATERIALS AND METHODS

### Test compounds.

Recombinant gp120 from HIV-1(III_B_), produced in CHO cells, and HIV-1(YU2), produced in insect cells, were purchased from Immunodiagnostics (Woburn, MA). α-1,2-Mannobiose and α-1,3-mannobiose were purchased from Dextra (United Kingdom). Laminin, from human placenta, and mucin, partially purified from porcine stomach, were purchased from Sigma, while BSA-GlcNAc was purchased from Dextra Laboratories (United Kingdom).

### Viruses, cell lines, and cell cultures.

HIV-1 R5 strain BaL and HIV-1 X4 strain NL4.3 were originally obtained through the AIDS Research and Reference Reagent Program (Division of AIDS, NIAID, NIH). MT-4 cells were a gift from L. Montagnier (during that time at the Pasteur Institute, Paris, France) and cultured in RPMI 1640 medium supplemented with 10% fetal calf serum (FCS; HyClone, Perbio Science) and 2 mM l-glutamine (Invitrogen) at 37°C in a 5% CO_2_ controlled atmosphere. PBMCs from healthy donors were isolated from buffy coats obtained from the Blood Transfusion Centre (UZ Leuven, Belgium). PBMCs were cultured in RPMI 1640 containing 10% FCS, 2 mM l-glutamine, and 2 ng/ml interleukin-2 (IL-2; Roche Molecular Biochemicals). The cells were activated with 2 µg/ml phytohemagglutinin (PHA; Sigma-Aldrich) for 3 days before infection with HIV-1. TZM-bl cells were obtained from the AIDS Research and Reference Reagent Program (Division of AIDS, NIAID, NIH).

### Expression and purification of FimH-LD, N-Als3p, N-Als1p, and N-Epa1p.

The sequence of FimH-LD from *E. coli* K-12 (strain K514) was used for this work (residues 22 to 179; UniProt entry P08191). The cloning, expression, and purification of FimH-LD have been previously described (10). The N-terminal parts of the Als3, Als1, and Epa1p proteins were expressed in and purified from *S. cerevisiae* and *E. coli* as previously described (22, 27).

### Glycan array screening.

The N-terminal parts of Als3p and Als1p were subjected to glycan array screening for binding to glycans printed on a glass slide microarray (version 5.0) developed by the CFG (5). Screening of Als3p was performed at concentrations of 20 and 200 µg/ml. The adhesins were labeled with NT-647 dye via an amine-coupling method (NanoTemper) (see the supplemental material).

### SPR.

SPR experiments were performed with a Biacore 3000 instrument (GE Healthcare) at 25°C. The recombinant gp120 envelope proteins, BSA-GlcNAc, FN, mucin, and laminin were covalently immobilized on a CM5 sensor chip by amine-coupling chemistry. A reference flow cell chemically treated in the same way as the ligand flow cell was used as a control. For FimH-LD–gp120 kinetic analysis, fitting of experimental curves and calculation of kinetic parameters were performed by using the BIAEvaluation software version 4.1 (GE Healthcare) and a 1:1 (Langmuir) binding model. In all of the other cases, dissociation constants in the equilibrium state (*K_D_*) were determined. The results were then analyzed with the BIAevaluation software and with Prism 6 software (GraphPad) (see the supplemental material).

### AFM-SMFS.

AFM-SMFS experiments to determine the unbinding force between HIV-1(YU2) gp120 and FimH-LD were performed as described in the supplemental material.

### LGI network construction.

The results of the glycan array screenings for FimH-LD (L. Wyns, 2011, CFG database, glycan array version 5.0) N-Als1p (first screening, glycan array version 3.2 [22]; second screening, CFG glycan array version 5.0 [this work]), N-Als3p, N-Epa1p (28), N-Epa6p, and N-Epa7p (85) were retrieved from the CFG-Core H database (http://www.functionalglycomics.org/) and used to generate the Als/Epa-glycan interaction networks. The results were filtered by removing the data of three times the standard error of the mean (SEM) and using a signal-to-noise ratio cutoff value that was larger than the average number of relative fluorescence units (RFU). The signal-to-noise ratio cutoff value was visually selected on the glycan array screening graphs. The RFU values were normalized by dividing the values by the maximal RFU value of the screening. The mean RFU value was used when more than one value was detected in different screenings for the same glycan interaction. Glycan-binding protein binding sites may accommodate glycan determinants made up of two to six linear monosaccharides together with their potential side chains containing other sugars and modifications (4). Therefore, glycan determinant structures containing oligosaccharides composed of 2, 3, 4, 5, and 6 carbohydrate residues present at the nonreducing end were submitted to the GlycoSuiteDB database in the UniCarbKB platform (46, 47).

In order to obtain the glycoproteomic data, we developed a set of three Perl scripts that collaborated to extract the data from the GlycosuiteDB website (see the supplemental material).

### LGI network modeling and visualization.

The interactions were modeled as a weighted undirected graph *G*(*V*, *E*, *W*), where *V* is the set of vertices, *E* is the set of edges connecting the pairs of vertices, and *W* is the set of weights associated with each edge. The edges’ weights were calculated according to the interaction type (see the supplemental material). The Gephi open-source graph visualization and manipulation software was used (86). Within Gephi, the force-directed visualization method of Hu (49), which is known to be accurate when visualizing local clustering and symmetry (87), was used. This approach allowed us to obtain relatively well-defined spatial distributions and local clusters according to the network structure (Fig. 2 and 5). The LGI networks were also visualized as a hierarchical structure by using Cytoscape 2.8 (88) and the Cerebral app (89) (see the supplemental material).

### MTS-PES antiviral assays.

The anti-HIV-1 activity of each compound, both alone and in combination, in MT-4 cell cultures was determined by a tetrazolium-based colorimetric assay. Briefly, 3-fold dilutions of the test compounds were added to a 96-well plate, and it was preincubated for 20 min at 37°C with MT-4 cells. Five days postinfection, CPEs were scored microscopically and antiviral activity was measured by the MTS-PES method with a SpectraMax 96-well plate reader (Molecular Devices) as described previously (90). PHA-stimulated PBMCs were resuspended in cell culture medium supplemented with 2 ng/ml IL-2 and seeded into 48-well plates (Iwaki Glass) containing various concentrations of test compounds. After 20 min of preincubation at 37°C, infection with HIV-1 was performed. IL-2 was added at days 3 and 6 postinfection. Supernatant was collected at day 10, and viral replication was measured with an HIV-1 p24 Ag ELISA (PerkinElmer) according to the manufacturer’s guidelines.

### TZM-bl–HIV-1 infectivity luminescence assay.

Firefly luciferase- and *E. coli* β-galactosidase-expressing CD4^+^ CXCR4^+^ CCR5^+^ TZM-bl cells were resuspended in cell culture medium (Dulbecco’s modified Eagle’s medium with 10% FCS and 1% HEPES) supplemented with 15 µg/ml DEAE-dextran (Sigma-Aldrich, Diegem, Belgium) and preincubated for 30 min at 37°C in cell culture medium-diluted test compounds in 96-well plates. Next, a laboratory HIV-1 strain (X4 NL4.3 or R5 BaL) was added according to the 50% tissue culture infective dose of the viral stock. Two days postinfection, CPEs were first scored microscopically, and afterward, viral replication was measured by luminescence. Steadylite plus reagent (PerkinElmer) was mixed with lyophilized substrate in accordance with the manufacturer’s guidelines. Supernatant was removed, and the steadylite plus substrate solution was added to the 96-well plates. Next, the plates were incubated in the dark for 10 min in a closed plate shaker. Finally, cell lysis was scored microscopically and aliquots were transferred to white Lumitrac 96-well plates (Greiner Bio-One) to measure the relative luminescence units with a SpectraMax L microplate reader and SoftMax Pro Software (Molecular Devices), an integration time of 0.6 s, and a dark adaptation time of 5 min.

## SUPPLEMENTAL MATERIAL

Text S1  Supplemental materials and methods. Download Text S1, DOCX file, 1.3 MB

Figure S1  Analysis of FimH-LD–HIV1(YU2) gp120 interactions by AFM-SMFS. (A) Force-distance retraction curves. The red and blue (middle and top) curves both show a typical bond rupture peak, while the black (bottom) curve is an example of a retraction curve without any unbinding event. (B) Representative adhesion event force histogram (green histogram) obtained from a set of 1,000 force-distance curves. The force intensities of the unbinding events detected are on the *x* axis, while the relative unbinding event frequencies are on the *y* axis. The inhibitory effect of 10 mM α-1,3-mannobiose on FimH-gp120 adhesion event distribution is shown in the red histogram. (C) Fitted value and related standard deviation of the single-molecule force distribution of the FimH-LD–gp120 interactions obtained from a set of 1,000 curves by using a classical Gaussian model. Download Figure S1, TIF file, 2.1 MB

Figure S2  Glycan array spectrum of N-Als3p and N-Als1p. (A to D) Determination of the glycan specificities and affinities of N-Als3p by glycan array screening. The N-Als3p glycan array screening graphs for concentrations of 200 (A) and 20 (C) µg/ml are shown. The glycan data analysis of N-Als3p and the distribution of the most prevalent ligands obtained for 200 (B) and 20 (D) µg/ml are shown. The structures for the highest scores are shown schematically. N-Als3p exhibits the strongest binding (concentration of 200 µg/liter) of multiple structures (up to six) of Galβ1-4GlcNAc carbohydrate (type 2 LacNAc), including biantennary complexes (A). Both types of complex glycans, with either terminal galactose or terminal GlcNAc, are bound, indicating no preference for a specific order of the components of the LacNAc glycan. Both α-2,3- and α-2,6-sialylated LacNAc forms are recognized. No preference for sulfated LacNAc is demonstrated. N-Als3p showed significant binding of poly(LacNAc), with internally fucosylated LacNAc units encompassing all Lewis antigens (Le^x^, Le^y^, Le^a^, Le^b^). N-Als3p bound also to the T antigen, which is a cancer-associated antigenic determinant (G. F. Springer, P. R. Desai, M. S. Murthy, H. Tegtmeyer and E. F. Scanlon, Prog Allergy 26:42–58, 1979; J. M. Rhodes, B. J. Campbell, and L.-G. Yu, Biochem Soc Trans 36:1482, 2008). Carbohydrates such as GlcNAcβ-1,3GalNAc and Fucα-1,2-Galβ-1,3-GalNAcβ-1,3-Gal were also recognized (marked as “other” in panel B). At a concentration of 20 µg/ml, N-Als3p shows a binding profile similar that of the same three top LacNAc binders but detected in a different order (C, D). The longest LacNAc glycan consists of nine repetitive units of LacNAc. Fewer glycans containing the Le^y^ antigen are found among the ligands, but there are more carbohydrates containing the T-antigen motif bound to N-Als3p at this concentration. The α-linked antigen GalNAc is the best-binding monosaccharide at the 200-µg/ml protein concentration (A, B), and the α-linked sulfated and nonsulfated forms of GalNAc are preferentially bound at 20 µg/ml (C, D). The lower protein concentration also binds to β- and α-linked, sulfated mannose and α-linked fucose, galactose, and glucuronic acid. (E) N-Als1p was labeled with the same dye to check the binding profile (compared to a previous glycan array analysis, where N-Als1p was labeled with a fluorescently labeled anti-His antibody [D. S. Donohue, F. S. Ielasi, K. V. Y. Goossens, and R. G. Willaert, Mol Microbiol 80:1667–1679, 2011]). The protein (189 µg/ml) was labeled with NT-647 dye via an amine-coupling method in accordance with the manufacturer’s instructions. The strongest binder was exactly the same poly(LacNAc) glycan that was bound the most strongly by N-Als3p. However, most hits showed a high coefficient of variation and were not considered for further analysis. Download Figure S2, TIF file, 0.9 MB

Table S1  Predicted potential glycoprotein binding receptors for *Candida* Als and Epa adhesins. Table S1, DOCX file, 0.1 MB

Table S2  Human proteins in the LGI network and related abbreviations. Table S2, DOCX file, 0.1 MB
