# Supplementary material for: Lectin-Glycan Interaction Network-Based Identification of Host Receptors of Microbial Pathogenic Adhesins
Source: mBio. 2016 Jul 12;7(4):e00584-16. doi: 10.1128/mBio.00584-16 (PMC4958244; doi:10.1128/mBio.00584-16)
Supplement: Text S1 — Supplemental materials and methods. Download [file mbo003162889s1.docx]

**Text S1: Supplemental Material**

**Supplemental Materials and Methods**

#### Expression and purification of N-Als3p, N-Als1p and N-Epa1p

The N-terminal parts of Als3 (18-432 aa) and Als1 (18-432 aa) proteins were expressed and purified as previously described (1). Briefly, the *In Fusion^TM^* method was used for homologous recombination between the vector and the amplified gene. The sequences of the oligonucleotides designed to amplify the *ALS3* gene with the C-terminal His-tag were 5’-TCCTTAGTCAAAAGGAAGACAATCACTGGTGTTTTTGATAGTTTTAATTCAT-3’ and 5’-GGAGATCGGAATTCGTCAGTGATGGTGATGGTGATGTGGAACTTGTACCACCATGTGT-3’. The plasmid pYEX-S1, which is a yeast-*Escherichia coli* shuttle vector with the full-length leader sequence from *Kluyveromyces lactis* was used to direct proteins through the secretory pathway. The S288C-derived BY4741 *S. cerevisiae* yeast strain has been used to transform plasmids carrying the *ALS3* gene. The N-Als3p constructs were purified by using Ni-NTA affinity chromatography and gel filtration chromatography using a Superdex 75 HR 10/30 column. Next, the purity of fractions was analyzed *via* sodium dodecyl sulphate polyacrylamide gel electrophoresis (SDS-PAGE).

Expression and purification of N-Epa1p from *E. coli* has been described (2). The N-Epa1p construct used in this work was re-cloned, in order to cover the same protein sequence of the construct previously described, except for the C-terminal motif CASVKTS (aa 272-278). This deletion avoided protein aggregation due to the formation of intermolecular disulfide bonds and did not modify the protein activity.

#### Glycan array screening

The N-terminal parts of Als3p (18-432 aa) and Als1p (18-432 aa) were subjected to glycan array screening for binding to glycans that were printed on a glass slide microarray (version 5.0). The screening of Als3p was performed at concentrations of 20 µg/ml and 200 µg/ml. The proteins were labeled with the NT-647 dye (excitation wavelength of 650 nm, emission wavelength of 670 nm) *via* an amine coupling method, according to manufacturer’s instructions (NanoTemper). The average relative fluorescence units were obtained for four replicates for each glycan. Error bars in Fig S2E are based on the standard error of the mean (SEM) for these replicates. The indicated intensity of N-Als3p (200 µg/ml) binding to different carbohydrates is with up to 3.5% fluorescent intensity of the highest binder. For comparison purposes, the highest hit for the Le^b^ was added (relative fluorescence unit value [RFU] was less than 3.5% intensity of the highest binder). The indicated intensity of Als3p-N (20 µg/ml) binding to different carbohydrates is with up to 10% fluorescent intensity of the highest binder. The hits with a very high SEM (≥ RFU) were not included. Error bars are based on the standard error of the mean (SEM) for four replicates. Glycan structures that are displayed on the graphs were drawn using The GlycanBuilder (3, 4).

### Surface Plasmon Resonance

SPR experiments were performed with a Biacore 3000 instrument (GE Healthcare) at 25°C. The recombinant HIV-1 (YU2) and HIV-1 (III_B_) gp120 envelope proteins, BSA-GlcNAc, fibronectin, mucin and laminin were covalently immobilized on a CM5 sensor chip by using amine-coupling chemistry. A reference flow cell, chemically treated in the same way as the ligand flow cell, was used as the control. FimH-Ld and N-Als3p solutions used for the analyses were prepared in HBS buffer (10 mM HEPES pH 7.4, 150 mM NaCl, 10 mM EDTA, 0.005% v/v Tween-20), while N-Epa1p solutions were diluted in 10 mM HEPES pH 7.4, 150 mM NaCl, 10 mM CaCl_2_, 0.005% v/v Tween-20. All protein solutions were injected at a flow-rate of 20 μl/min. The sensor chip was regenerated with 5 mM NaOH, in the case of FimH-Ld and N-Epa1p analyses, or, with 0.1% w/v sodium dodecyl sulfate (SDS), in the case of the N-Als – fibronectin analysis, while no regeneration step was required for the other N-Als3p analyses. For FimH-Ld – gp120 kinetic analysis, gp120 was immobilized on the CM5 sensor chip, and FimH-Ld solutions were prepared by 2-fold serial dilutions in a concentration range from 60 μM to 60 nM. The fitting of the experimental curves and calculation of the kinetic parameters were performed by using the BIAEvaluation software version 4.1 (GE Healthcare), and a 1:1 (Langmuir) binding model. In all other cases, dissociation constants at the equilibrium state (*K_D_*) were determined by measuring the increase in resonance units after subtraction of the background response obtained from the reference flow cell, and the sample containing only the buffer. *K_D_* values were estimated using a one-binding site model, with the formula: *R_eq_* = *R_max_* (*K_A_C_A_*)/(*K_A_C_A_*+1), where *R_eq_* is the response at the equilibrium state, *K_A_* is the association constant at the equilibrium state, *C_A_* is the analyte concentration, and *R_max_* is the maximum binding capacity. The results were analyzed with the BIAevaluation software and with Prism 6 (GraphPad) software. For the Fimh-Ld – gp120 inhibition experiments, two disaccharides (Manα-1,2-Man and Manα-1,3-Man) that mimic different moieties on the viral protein glycosylation sites, were used. Samples with a fixed concentration of FimH-Ld (8 μM) and varying carbohydrate concentrations were injected over the sensor chip. *R_eq_* values from the sensorgrams were used to calculate inhibition constants.

**Atomic Force Microscopy – Single Molecule Force Spectroscopy**

The FimH-Ld substrate for the force spectroscopy measurements was prepared by treating a freshly cleaved mica surface first with 1% v/v 3-aminopropyltrietoxysilane (APTES) acqueous solution for 1 min, then with 1% v/v gluteraldehyde acqueous solution for 1 h (5). Subsequently, mica was incubated with a 1 mg ml^-1^ protein solution in 20 mM Tris-HCl buffer pH 8 150 mM NaCl for 1 h. Unbound aldehyde groups on the surface were blocked with 10 mM Tris-HCl pH 7.4 for 1 h. AFM tips functionalized with gp120 - HIV-1(YU2) were prepared by treating silicon nitride DNP-10 cantilevers (Bruker) with 1% v/v gluteraldehyde acqueous solution for 1 h, followed by an incubation step with 1 mg ml^-1^ protein solution in PBS for 1 h, and subsequent blocking with Tris-HCl. Force measurements were performed using a Nanoscope IIIa Multimode AFM, equipped with a Picoforce scanner (Bruker).

All measurements were performed at room temperature in a liquid cell filled with protein buffer. Tips with nominal spring constants, *k,* of 0.06 N/m were selected for the analyses. The effective spring constant of the cantilevers were measured using the thermal noise method, and were found to be 0.113 N/m. A constant approach and retraction speed of 969 nm/s was used during all measurements. Inhibition experiments were performed by injecting a 10 mM α-1,3-mannobiose solution in the protein buffer into the AFM liquid cell. After 30 min of incubation at room temperature, force measurements were performed in the same conditions. Three sets of thousand force-distance curves were acquired for all experiments. Unbinding rupture events were detected as peaks on the retraction curves, and evaluated by measuring the height of the peaks from the baseline. Detection of peaks was performed using the Open Fovea software (6), and protein-protein unbinding event distributions were reported as adhesion force *versus* event frequency distributions, using the Origin software (OriginLab Corp.). Fitting of the unbinding event distributions was performed with the same program, using a classical Gaussian model.

**Modeling and visualizing lectin-glycan interaction (LGI) networks**

*Lectin-glycan interaction network definition*

The interactions network has been defined as a weighted undirected graph *G = (V, E)*, being *V* a set of nodes (vertices) and *E* a set of edges connecting pair of vertices:

- The vertices *V* represent the elements of the network: *Glycan Determinants, Proteins, Diseases, Human Tissues, Human Systems, Protein Types, and Protein Classes*. Depending on the interactions subject to study, the resultant network can include all or a subset of the mentioned elements. For each node we associate a relevance as defined below (“*Node Relevance*”).
- The edges *E*, can represent biochemical interactions, biological and/or hierarchical associations between the nodes. For each edge we associate a weight, according to the interactions type sustaining it:

1. *Glycan Determinant* to *Protein*: this relationship represents a biochemical interaction associated to a set of lectin binding intensity values, from which the weight *W_g,p_* is computed according to Eq. (1).
2. *Disease* to *Glycan Determinant* and/or *Protein*: this relationship represents a biological association of certain disease resulting from a several glycan determinants and proteins interactions. The corresponding set of lectin binding intensity values associated to these interactions will provide the final value of *W_g,d_ (or W_p,d_)*, according to Eq. (2) (or Eq.(3)).
3. For any other relationship the associated weight, *Wi,j,* (of the edge connected a node *i* to a node *j*) is a constant value, set to *W_i,j_*=*0.5* in all our experiments.

*Proposed edges relevance and* lectin binding intensity

The proposed weights *W* are closely related to the biological meaning of the relationships between any two nodes. In the case of *Glycan Determinant*, *Protein* and *Diseases* they correspond to the experimentally measured lectin binding intensity. Since several measurements have been performed per interaction (see Results, section “Modeling and visualization of lectin-glycan interaction (LGI) networks”), the mean of all measurements provides the edge relevance:

|  | $W_{g,p}=\lambda\left[\sum_{d\in\emptyset\cup\{Diseases\}} \frac{1}{M}\sum_{m=1}^{M} f_{m}(g,p\vert d)\right]$ | (1) |
| --- | --- | --- |

where *f_m_*() is an experimental measurement of the binding intensity between glycan *g* and protein *p*, having the disease *d* associated (or without any association at all), and *M* is the total of those measurements. The λ factor plays a re-scaling role (i.e. normalization) mostly for visualization purposes. It is a fixed value for all the edges per network analysis and visualization result. In the case a *Disease*, the edge relevance is defined as follows:

|  | $W_{g,d}=\max_{p\in\{Proteins\}} \lambda\left[\frac{1}{M}\sum_{m=1}^{M} f_{m}(g,p\vert d)\right]$ | (2) |
| --- | --- | --- |
|  | $W_{p,d}=\max_{g\in\{Glycans\}} \lambda\left[\frac{1}{M}\sum_{m=1}^{M} f_{m}(g,p\vert d)\right]$ | (3) |

Fig. SMM1 illustrates the weights associated to the edges connected to a *Disease* node.


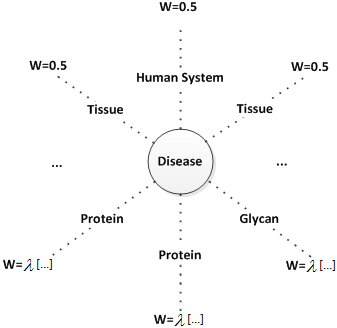


**FIG SMM1**: Example of a *Disease* node. Edge values are defined according to the type of interaction.

*Node Relevance*

Determining the relevance of a node is a known problem in network analysis, and can be solved via node’s centrality measures. As the modeled LGI is a weighted graph, we opted to follow a variant of the PageRank approach (7) where both the node neighborhood and the associated weights are taken into account to determine the node’s centrality. The PageRank algorithm (7) involves each node neighborhood and also the associated edges weights to estimate the rank of a node. The ranking *PR(i)* of the *i*-th node is defined as:

$PR(i)=\frac{1-d}{N}+d\left(\sum_{j\in\eta(i)} \frac{PR(j)W_{i,j}}{\sum_{k\in\eta(j)} W_{k,j}}\right)$ (4)

where *N* is the number of nodes in the graph, *η(v)* represents the neighbors of a node *v*, and *d* is a damping factor set to 0.99 in our experiments, the edge values *W_i,j_* and *W_k,j_* are as defined in Eqs. (1), (2) and (3), depending on the type of the connecting nodes (*i*, *j*). The PageRank computation procedure starts with a *PR(.) = 1/N* for each node of the graph. Then an iterative process updates all the nodes once per iteration, until the nodes do not change values between two consecutive iterations (*PR(.)* obtained in two consecutive iterations is less or equal than *ξ* = 10^-3^). Since the centrality value *PR(.)* of a node involves the normalized sum of all its connected edges, the re-scaling factor λ (of Eqs. 1-3) allows highlighting different type of information. For instance, if λ is close to zero, the centrality measure will mainly depend on the number of edges connected to the particular node; in the other hand, if λ is high, the centrality will be mostly determined by the measured binding intensity of the biochemical interaction. Intermediate values of λ will regulate the importance of both type of information (number of edges and measured binding intensity) for the final estimate of the node’s centrality measure.

Fig. 5 illustrates a network where the centrality has been computed using very low values (Panel A) and very high values of λ (Panel B), respectively. In Fig. 5A, the nodes related to *Human Tissues* are the most important since they are the most connected ones, while in Fig. 5B those *Diseases* and *Proteins* that exhibited the highest binding intensity (thickness in arrows are proportional to λ and binding intensity values) are the ones with higher centrality.

**
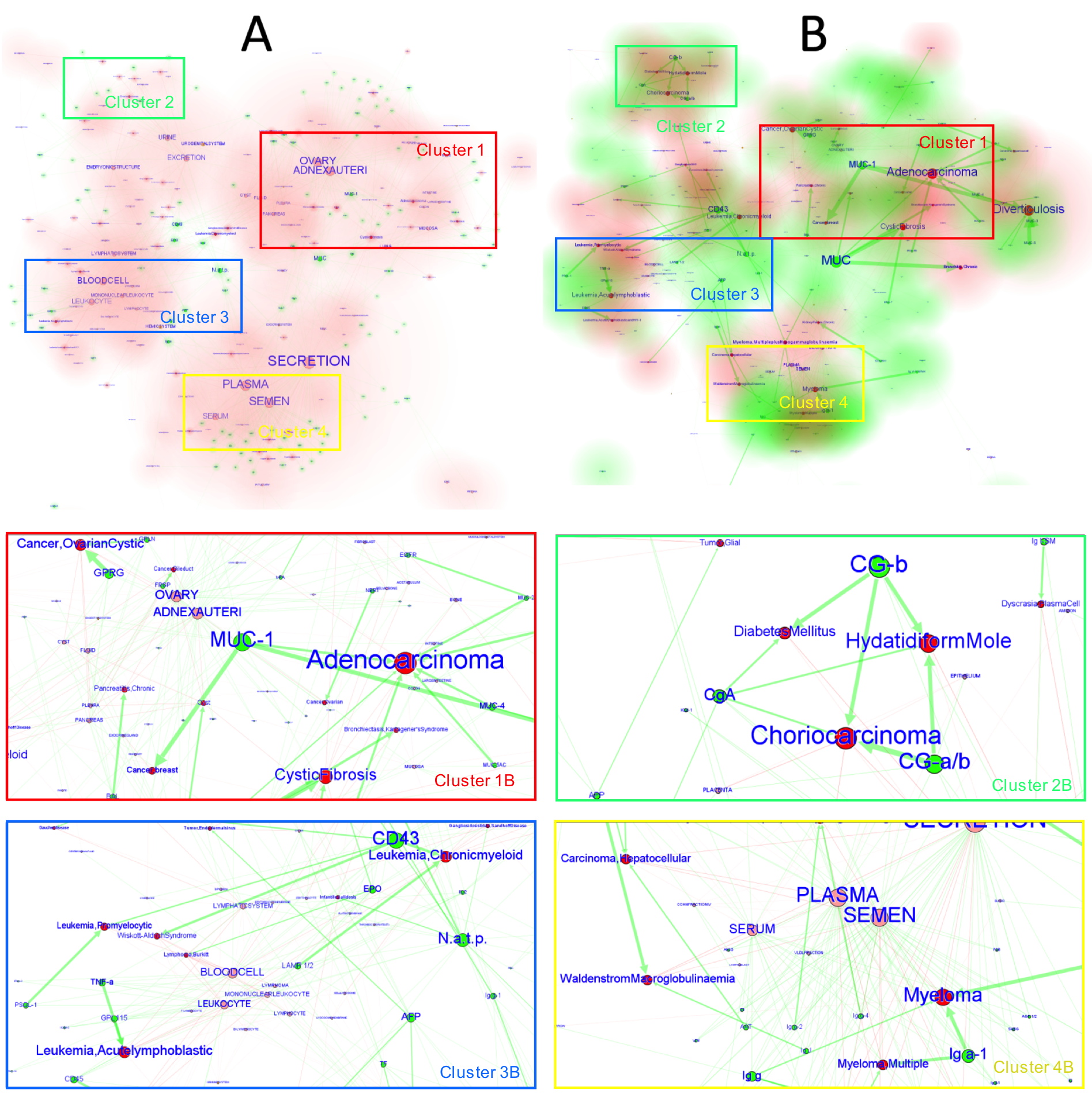
**

**FIG 5** Different values for λ highlight different information present within the same network (see the main text for the detailed figure caption).

*Visualization*

Several graph visualization methods can be found in the literature (8). In this work, we selected a force-directed method proposed by Hu (9) The model is based on the definition of an attractive force (*Fa*) and a repulsive force (*Fr*) between vertices, which acts (inversely) proportional to the distance between them, defined as follows:

$$F_{a}\left( i,j,K \right)=\left( i-j \right).\left\| i-j \right\|^{2}/K$$

$$F_{r}\left( i,j,C,K \right)=C.K^{2}.(i-j)/\left\| i-j \right\|^{2}$$

where, *i*, *j* are two nodes, *K* is a parameter known as “optimal distance”, and *C* is a parameter regulating the relative intensity of repulsive forces between the nodes (fixed to *C*=0.2 in all the experiments). The value of *K* is manually adjusted depending on the size of the graph, and have an impact in the total area covered by the visualization. The final layout of the visualization is obtained by minimizing an energy functional, involving the forces *Fa* and *Fr*, estimated over all the nodes of the graph, (see Algorithm 1 in (9) for more details). To visualize the graph, we make use of the Gephi toolkit (It is an early but useful open source java toolkit for visualizing general networks, and is freely available at http://www.gephi.org/), and benefited from the Hu algorithm implementation available on the same package. The result showed relatively well defined spatial distributions of clusters according to the network structure, whenever these clusters existed, as illustrated in Fig. 5 and Fig. SMM2.


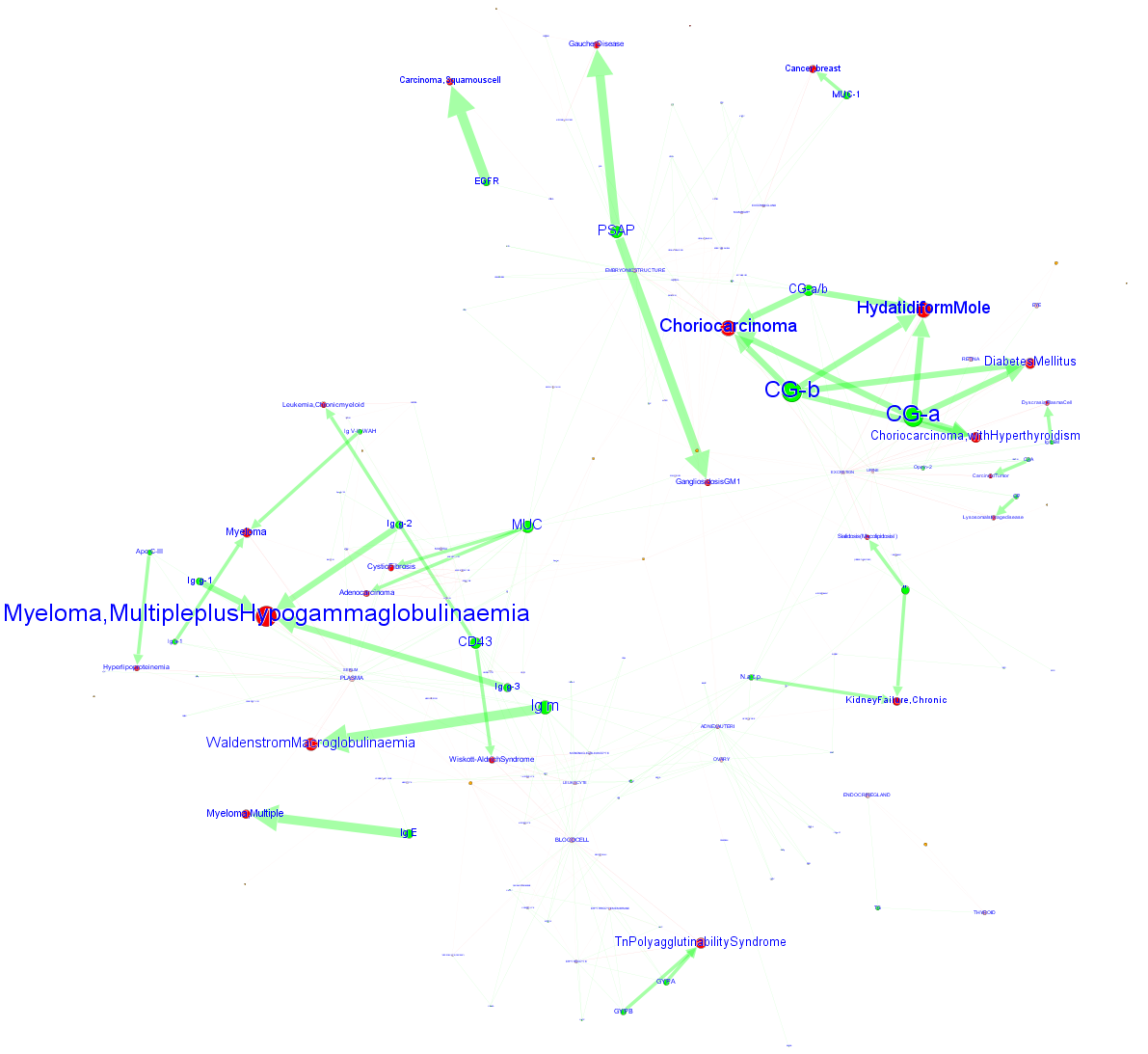


**FIG SMM2** Clusters are revealed after minimization of the system energy F***(x,C,K)***.

**Hierarchical LGI network graphs using the Cerebral app in Cytoscape**

Cerebral (Cell Region-Based Rendering And Layout) (10) is an open-source Java plugin for the Cytoscape network viewer. Cerebral automatically generates a view of the network in the style of traditional pathway diagrams with subcellular localization annotation, providing an intuitive interface for the exploration of a biological pathway or system. We used Cerebral to create hierarchical views of the LGI networks (Fig 1A, 3, and 7A): lectins were related to the bound glycan molecules in the glycan array, the glycan determinants, the human or viral glycoproteins found in the glycoproteomics database, the localization of the human glycoproteins in the human tissues and their corresponding body systems.

**The Parser scripts and LGI network algorithm**

A set of three Perl scripts that collaborate to extract the data from the GlycosuiteDB website were developed. Firstly, a manual query was performed on the http://www.unicarbkb.org/builder webpage, for each of the glycan determinants obtained during the glycan screening stage, saving each time the resultant webpage in .html format. In our case, we generate 8 files (query1.html, …, query8.html). Secondly, a script “get_structures.pl” processed each of the above 8 files, extracting the necessary information on each entry and automatically querying the http://www.unicarbkb.org/structure/ webpage (471 times in our case) to generate a set of structure-related entries. Thirdly, for each of the entries obtained in the previous step, the script “parse_proteins.pl” automatically queried the http://www.unicarbkb.org/proteinsummary/ webpage (200 times in our case) looking for proteins associated to the glycan-determinant in question, and generate a set of proteins-related files. Finally, the “parse_structures.pl” extracts the data filtered to human-related diseases only, and save the results as an Excel file to enhance each entry. All the parser scripts are provided, along with a set of instructions on how to use them to repeat the experiments made in the manuscript or to perform new ones.

These can be downloaded from the dedicated website: http://www.etro.vub.ac.be/RESEARCH/AVSP/Downloads/. The Parser scripts and the results of each step during the paring process are available. Step-by-step instructions on how to use the scripts are provided (README.txt)s. The LGI Network algorithm (GlycanNetwork_0.95_release.jar), the input files, to generate the Als (Als.csv), Epa (Epa.csv), Als-Epa (Candida_Epa_PLUS_Als_network.csv), and FimH (FimH_network.csv) networks are available as well as step-by-step instructions to run the program (UsersGuide).

**Supplemental Materials and Methods References**

1. **Donohue DS**, **Ielasi FS**, **Goossens KVY**, **Willaert RG**. 2011. The N-terminal part of Als1 protein from *Candida albicans* specifically binds fucose-containing glycans. Mol Microbiol **80**:1667–1679.

2. **Ielasi FS**, **Decanniere K**, **Willaert RG**. 2012. The epithelial adhesin 1 (Epa1p) from the human-pathogenic yeast *Candida glabrata*: structural and functional study of the carbohydrate-binding domain. Acta Crystallogr D Biol Crystallogr **68**:210–217.

3. **Damerell D**, **Ceroni A**, **Maass K**, **Ranzinger R**, **Dell A**, **Haslam SM**. The GlycanBuilder and GlycoWorkbench glycoinformatics tools: updates and new developments. Biological Chemistry **393**.

4. **Ceroni A**, **Dell A**, **Haslam SM**. 2007. The GlycanBuilder: a fast, intuitive and flexible software tool for building and displaying glycan structures. Source Code for Biology and Medicine (SCFBM) **2**:3.

5. **Yersin A**, **Hirling H**, **Steiner P**, **Magnin S**, **Regazzi R**, **Hüni B**, **Huguenot P**, **De Los Rios P**, **Dietler G**, **Catsicas S**, **Kasas S**. 2003. Interactions between synaptic vesicle fusion proteins explored by atomic force microscopy. Proc Natl Acad Sci USA **100**:8736–8741.

6. **Roduit C**, **Saha B**, **Alonso-Sarduy L**, **Volterra A**, **Dietler G**, **Kasas S**. 2012. OpenFovea: open-source AFM data processing software. Nat Meth **9**:774–775.

7. **Iván G**, **Grolmusz V**. 2011. When the Web meets the cell: using personalized PageRank for analyzing protein interaction networks. Bioinformatics **27**:405–407.

8. **Agapito G**, **Pietro Hiram Guzzi**, **Cannataro M**. 2013. Visualization of protein interaction networks: problems and solutions. BMC Bioinformatics **14 Suppl 1**:S1–S1.

9. **Hu Y**. 2005. Efficient, high-quality force-directed graph drawing. Mathematica Journal **10**:1.

10. **Barsky A**, **Gardy JL**, **Hancock REW**, **Munzner T**. 2007. Cerebral: a Cytoscape plugin for layout of and interaction with biological networks using subcellular localization annotation. Bioinformatics **23**:1040–1042.
