# Supplementary figures and images for: Lectin-Glycan Interaction Network-Based Identification of Host Receptors of Microbial Pathogenic Adhesins
Source: mBio. 2016 Jul 12;7(4):e00584-16. doi: 10.1128/mBio.00584-16 (PMC4958244; doi:10.1128/mBio.00584-16)

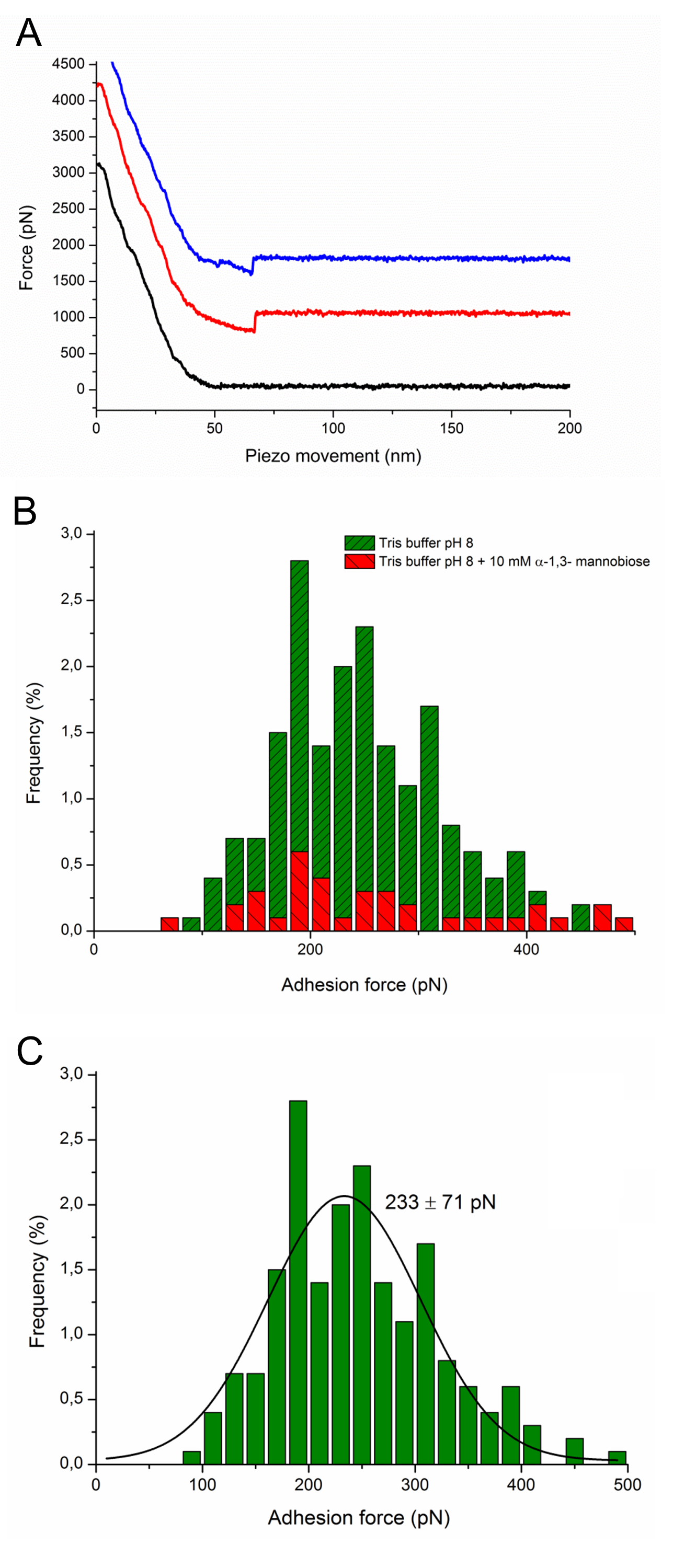

Supplement: Figure S1 — Analysis of FimH-LD–HIV1(YU2) gp120 interactions by AFM-SMFS. (A) Force-distance retraction curves. The red and blue (middle and top) curves both show a typical bond rupture peak, while the black (bottom) curve is an example of a retraction curve without any unbinding event. (B) Representative adhesion event force histogram (green histogram) obtained from a set of 1,000 force-distance curves. The force intensities of the unbinding events detected are on the x axis, while the relative unbinding event frequencies are on the y axis. The inhibitory effect of 10 mM α-1,3-mannobiose on FimH-gp120 adhesion event distribution is shown in the red histogram. (C) Fitted value and related standard deviation of the single-molecule force distribution of the FimH-LD–gp120 interactions obtained from a set of 1,000 curves by using a classical Gaussian model. Download [file mbo003162889sf1.tif]

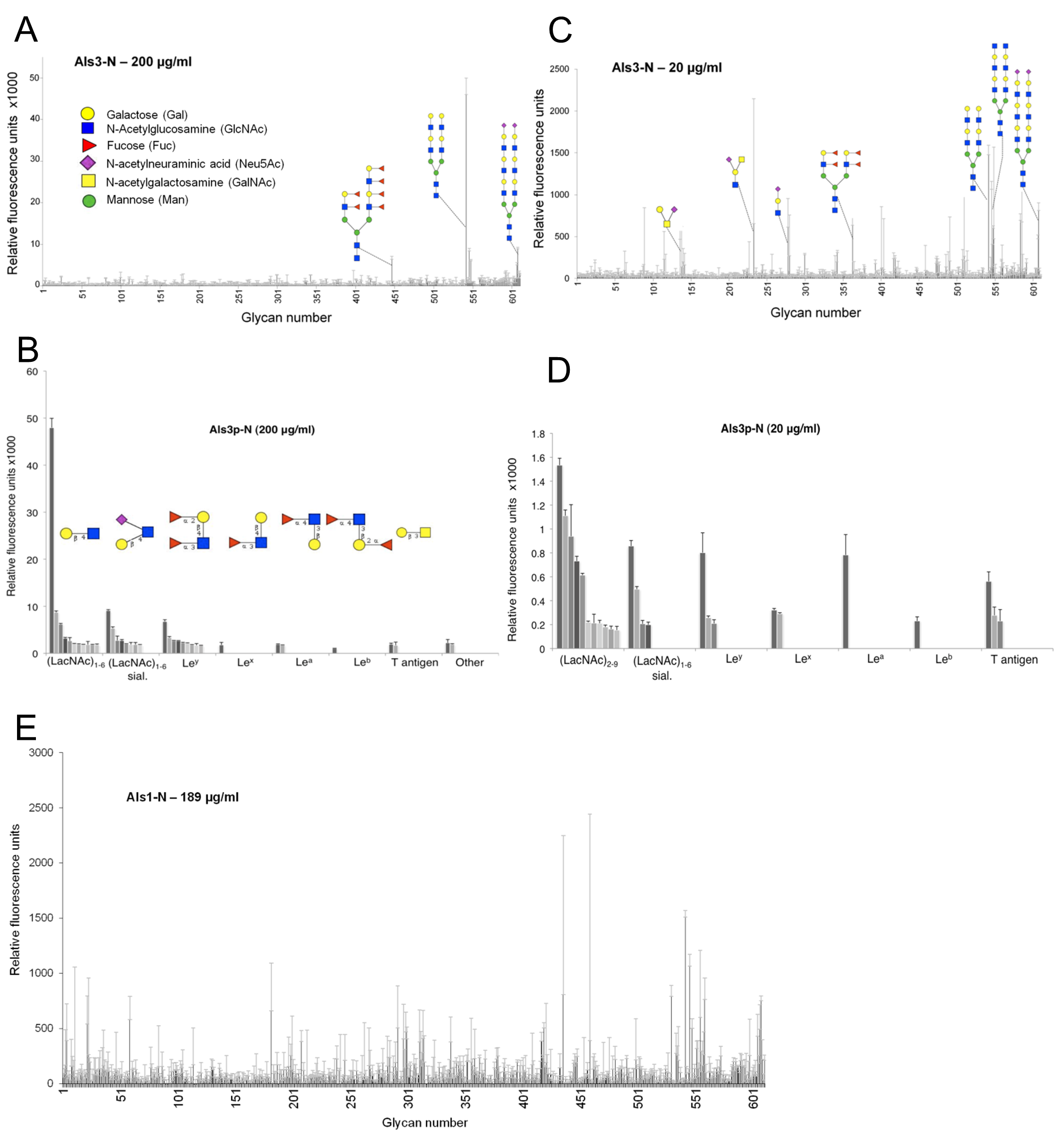

Supplement: Figure S2 — Glycan array spectrum of N-Als3p and N-Als1p. (A to D) Determination of the glycan specificities and affinities of N-Als3p by glycan array screening. The N-Als3p glycan array screening graphs for concentrations of 200 (A) and 20 (C) µg/ml are shown. The glycan data analysis of N-Als3p and the distribution of the most prevalent ligands obtained for 200 (B) and 20 (D) µg/ml are shown. The structures for the highest scores are shown schematically. N-Als3p exhibits the strongest binding (concentration of 200 µg/liter) of multiple structures (up to six) of Galβ1-4GlcNAc carbohydrate (type 2 LacNAc), including biantennary complexes (A). Both types of complex glycans, with either terminal galactose or terminal GlcNAc, are bound, indicating no preference for a specific order of the components of the LacNAc glycan. Both α-2,3- and α-2,6-sialylated LacNAc forms are recognized. No preference for sulfated LacNAc is demonstrated. N-Als3p showed significant binding of poly(LacNAc), with internally fucosylated LacNAc units encompassing all Lewis antigens (Lex, Ley, Lea, Leb). N-Als3p bound also to the T antigen, which is a cancer-associated antigenic determinant (G. F. Springer, P. R. Desai, M. S. Murthy, H. Tegtmeyer and E. F. Scanlon, Prog Allergy 26:42–58, 1979; J. M. Rhodes, B. J. Campbell, and L.-G. Yu, Biochem Soc Trans 36:1482, 2008). Carbohydrates such as GlcNAcβ-1,3GalNAc and Fucα-1,2-Galβ-1,3-GalNAcβ-1,3-Gal were also recognized (marked as “other” in panel B). At a concentration of 20 µg/ml, N-Als3p shows a binding profile similar that of the same three top LacNAc binders but detected in a different order (C, D). The longest LacNAc glycan consists of nine repetitive units of LacNAc. Fewer glycans containing the Ley antigen are found among the ligands, but there are more carbohydrates containing the T-antigen motif bound to N-Als3p at this concentration. The α-linked antigen GalNAc is the best-binding monosaccharide at the 200-µg/ml protein concentration (A, B), and the α [file mbo003162889sf2.tif]
