# Supplementary material for: Lectin-Glycan Interaction Network-Based Identification of Host Receptors of Microbial Pathogenic Adhesins
Source: mBio. 2016 Jul 12;7(4):e00584-16. doi: 10.1128/mBio.00584-16 (PMC4958244; doi:10.1128/mBio.00584-16)
Supplement: Table S1 — Predicted potential glycoprotein binding receptors for Candida Als and Epa adhesins. [file mbo003162889st1.docx]

**TABLE S1** List of predicted potential glycoprotein binding receptors for *Candida* Als and Epa adhesins. A list of abbreviations is available in Table S2 (supplemental material).

| Adhesin family | Predicted receptor | Comments | References |
| --- | --- | --- | --- |
| Als, Epa | MUC (several proteins) | N-Epa1p binding to mucin confirmed.  *C. albicans* adhesion to salivary mucin reported.  *C. albicans* adhesion to intestinal mucin and inhibitory effect of mucin to epithelial cell adhesion reported. | This work  (1)  (2) |
| Epa | TNF-α | Tumor necrosis factor production induced on human PBMCs by Epa1p-expressing *S. cerevisiae.* | (3) |
| Als, Epa | CD43/45 | Both glycoproteins potentially mediating *C. glabrata* adhesion to human leukemia cells and PBMCs. | (3-5) |
| Epa | LAMP1/2 | Potentially involved in the *C. glabrata* inhibition of phagolysosome formation in macrophages. | (6) |
| Als, Epa | CD144 | N-Als3p binding to cadherins confirmed. | (7) |
| Epa | BACE-1 / vWF | Glycoproteins associated to healthy kidney and brain tissues and bearing branched N-glycan structures specifically recognized by Epa1p and Epa7p but not Epa6p.  Epa1p/Epa7p adhesion to human brain and renal endothelial cells described; no adhesion of Epa6p. | (8) |
| Epa | FN | N-Epa1p binding to fibronectin confirmed. | (9) |
| Als, Epa | PLG / t-PA | Plasminogen bound by pathogenic yeasts (e.g. *Cryptococcus neoformans*) and used for blood brain barrier penetration and invasion of the central nervous system.  Rare *Candida* brain infection reported. | (10)  (11) |
| Als, Epa | EGFR | Interaction with Als3p described. | (12) |
| Epa | CP | Inhibitory effect of a Cohn fraction IV component on *C. glabrata* growth. | (13) |
| Als, Epa | TF | Transferrin inhibitory effect on *C. glabrata* growth.  *C. albicans* able to acquire iron from transferrin  *C. albicans* iron acquisition from ferritin mediated by Als proteins. | (13)  (14)  (15) |
| Als, Epa | LTF | Lactotransferrin formulation inhibiting *C. glabrata* adhesion on vaginal epithelial cells. | (16) |
| Als | CSPG4 | *C. albicans* binding to glycosaminoglycans (included chondroitin sulfate). | (17) |
| Als, Epa | κ-casein | Als5p binding to casein. | (18) |
| Als, Epa | CG-a/b | *C. albicans* binding to human choriogonadotropin. | (19) |

**Supplemental Table S1 References**

1. **Hoffman MP**, **Haidaris CG**. 1993. Analysis of *Candida albicans* adhesion to salivary mucin. Infect Immun **61**:1940–1949.

2. **de Repentigny L**, **Aumont F**, **Bernard K**, **Belhumeur P**. 2000. Characterization of binding of *Candida albicans* to small intestinal mucin and its role in adherence to mucosal epithelial cells. Infect Immun **68**:3172–3179.

3. **Kuhn DM**, **Vyas VK**. 2012. The *Candida glabrata* adhesin Epa1p causes adhesion, phagocytosis, and cytokine secretion by innate immune cells. FEMS Yeast Res **12**:398–414.

4. **Ruhl S**, **Cisar JO**, **Sandberg AL**. 2000. Identification of polymorphonuclear leukocyte and HL-60 cell receptors for adhesins of *Streptococcus gordonii* and *Actinomyces naeslundii*. Infect Immun **68**:6346–6354.

5. **Baba M**, **Yong Ma B**, **Nonaka M**, **Matsuishi Y**, **Hirano M**, **Nakamura N**, **Kawasaki N**, **Kawasaki N**, **Kawasaki T**. 2007. Glycosylation-dependent interaction of Jacalin with CD45 induces T lymphocyte activation and Th1/Th2 cytokine secretion. J Leukoc Biol **81**:1002–1011.

6. **Seider K**, **Brunke S**, **Schild L**, **Jablonowski N**, **Wilson D**, **Majer O**, **Barz D**, **Haas A**, **Kuchler K**, **Schaller M**, **Hube B**. 2011. The facultative intracellular pathogen *Candida glabrata* subverts macrophage cytokine production and phagolysosome maturation. J Immunol **187**:3072–3086.

7. **Phan QT**, **Myers CL**, **Fu Y**, **Sheppard DC**, **Yeaman MR**, **Welch WH**, **Ibrahim AS**, **Edwards JE Jr**, **Filler SG**. 2007. Als3 is a *Candida albicans* invasin that binds to cadherins and induces endocytosis by host cells. Plos Biol **5**:e64.

8. **Zupancic ML**, **Frieman M**, **Smith D**, **Alvarez RA**, **Cummings RD**, **Cormack BP**. 2008. Glycan microarray analysis of *Candida glabrata* adhesin ligand specificity. Mol Microbiol **68**:547–559.

9. **Ielasi FS**, **Verhaeghe T**, **Desmet T**, **Willaert RG**. 2014. Engineering the carbohydrate-binding site of Epa1p from *Candida glabrata*: generation of adhesin mutants with different carbohydrate specificity. Glycobiology **24**:1312–1322.

10. **Stie J**, **Bruni G**, **Fox D**. 2009. Surface-associated plasminogen binding of *Cryptococcus neoformans* promotes extracellular matrix invasion. PLoS One **4**:e5780.

11. **Fennelly AM**, **Slenker AK**, **Murphy LC**, **Moussouttas M**, **DeSimone JA**. 2013. *Candida* cerebral abscesses: a case report and review of the literature. Med Mycol **51**:779–784.

12. **Zhu W**, **Phan QT**, **Boontheung P**, **Solis NV**, **Loo JA**, **Filler SG**. 2012. EGFR and HER2 receptor kinase signaling mediate epithelial cell invasion by *Candida albicans* during oropharyngeal infection. Proc Natl Acad Sci U S A **109**:14194–14199.

13. **Petrou MA**, **Rogers TR**. 1988. The inhibitory effect of serum on the growth of *Torulopsis glabrata*. Int J Med Microbiol **25**:213–220.

14. **Knight SAB**, **Vilaire G**, **Lesuisse E**, **Dancis A**. 2005. Iron acquisition from transferrin by *Candida albicans* depends on the reductive pathway. Infect Immun **73**:5482–5492.

15. **Almeida RS**, **Brunke S**, **Albrecht A**, **Thewes S**, **Laue M**, **Edwards JE**, **Filler SG**, **Hube B**. 2008. The hyphal-associated adhesin and invasin Als3 of *Candida albicans* mediates iron acquisition from host ferritin. PLoS Pathog **4**:e1000217.

16. **Naidu AS**, **Chen J**, **Martinez C**, **Tulpinski J**, **Pal BK**, **Fowler RS**. 2004. Activated lactoferrin's ability to inhibit *Candida* growth and block yeast adhesion to the vaginal epithelial monolayer. J Reprod Med **49**:859–866.

17. **Klotz SA**. 1990. Adherence of *Candida albicans* to components of the subendothelial extracellular matrix. FEMS Microbiology Letters **68**:249–254.

18. **Gaur NK**, **Smith RL**, **Klotz SA**. 2002. *Candida albicans* and *Saccharomyces cerevisiae* expressing *ALA1/ALS5* adhere to accessible threonine, serine, or alanine patches. Cell Commun Adhes **9**:45–57.

19. **Bramley TA**, **Menzies GS**, **Williams RJ**, **Adams DJ**, **Kinsman OS**. 1990. Specific, high-affinity binding sites for human luteinizing hormone (hLH) and human chorionic gonadotrophin (hCG) in *Candida* species. Bioch Biophys Res Commun **167**:1050–1056.
