# Supplementary material for: Lectin-Glycan Interaction Network-Based Identification of Host Receptors of Microbial Pathogenic Adhesins
Source: mBio. 2016 Jul 12;7(4):e00584-16. doi: 10.1128/mBio.00584-16 (PMC4958244; doi:10.1128/mBio.00584-16)
Supplement: Table S2 — Human proteins in the LGI network and related abbreviations. [file mbo003162889st2.docx]

**TABLE S2** List of the human proteins in the lectin-glycan interaction network and the related abbreviations.

| **Human body system** | **Protein - full name** | **Protein abbreviation** |
| --- | --- | --- |
| Cardiovascular system | Chondroitin sulfate proteoglycan 4 | CSPG4 |
| Digestive system | Bile-salt-activated lipase | BAL |
|  | Chymotrypsin-like elastase family member 3B | ELA3B |
|  | Fucose-rich glycopeptides | FRGP |
|  | Mucin-1 | MUC-1 |
|  | Mucin-2 | MUC-2 |
|  | Mucin-3 | MUC-3 |
|  | Mucin-4 | MUC-4 |
|  | Mucin-5 | MUC-5 |
|  | Mucin-5AC | MUC-5AC |
|  | Mucin-5B (MG1) | MUC-5B |
|  | Mucin-6 | MUC-6 |
|  | Nonsecretory ribonuclease | Rnase 2 |
|  | Not attached to protein | N.a.t.p. |
|  | Proactivator polypeptide | PSAP |
|  | RGM | RGM |
| Endocrine system | Glycoprotein hormones alpha chain | FSH-a |
|  | Lutropin subunit beta | LH-B |
|  | Thyroglobulin | TG |
| Exocrine system | Alpha-amylase 1 | AMY1 |
|  | Immunoglobulin alpha | Ig a |
|  | Kappa casein | k-casein |
|  | Lactotransferrin (lactoferrin) | LTF |
|  | Mucin | MUC |
|  | Mucin-1 | MUC-1 |
|  | Mucin-5B (MG1) | MUC-5B |
|  | Mucin-7 (MG2) | MUC-7 |
|  | Not attached to protein | N.a.t.p. |
|  | Polymeric immunoglobulin receptor | PIgR |
|  | Proteoglycan | PRG |
|  | Tissue-type plasminogen activator | t-PA |
| Hemic system | Alpha-1-acid glycoprotein 1/2 | AGP 1/2 |
|  | Alpha-1-antichymotrypsin | ACT |
|  | Alpha-1-antitrypsin | AAT |
|  | Alpha-2-HS-glycoprotein | AHSG |
|  | Apolipoprotein (a) | Apo(a) |
|  | Apolipoprotein B-100 | Apo B-100 |
|  | Apolipoprotein C-III | Apo C-III |
|  | Ceruloplasmin | CP |
|  | Coagulation factor IX | F9 |
|  | Coagulation factor X | F10 |
|  | Collagen alpha-1(XVIII) chain (endostatin) | COL18A1 |
|  | Complement C3 | C3 |
|  | Fibrinogen | FG |
|  | Fibrinogen alpha chain | FGA |
|  | Fibrinogen beta chain | FGB |
|  | Fibronectin | FN |
|  | Glycophorin-A | GYPA |
|  | Glycophorin-B | GYPB |
|  | Glycophorin-C | GYPC |
|  | Immunoglobulin A1 | Ig A1 |
|  | Immunoglobulin alpha-1 | Ig a-1 |
|  | Immunoglobulin E | Ig E |
|  | Immunoglobulin gamma | Ig g |
|  | Immunoglobulin gamma-1 | Ig g-1 |
|  | Immunoglobulin gamma-2 | Ig g-2 |
|  | Immunoglobulin gamma-3 | Ig g-3 |
|  | Immunoglobulin gamma-4 | Ig g-4 |
|  | Immunoglobulin heavy chain V-II region WAH | Ig V-II WAH |
|  | Immunoglobulin J chain | Ig J |
|  | Immunoglobulin mu | Ig m |
|  | Immunoglobulin M | Ig M |
|  | Intercellular adhesion molecule 3 | ICAM-3 |
|  | Interferon alpha-14 | IFN-a-14 |
|  | Interferon alpha-2 | IFN-a-2 |
|  | Leukosialin | CD43 |
|  | Lysosome-associated membrane glycoprotein 1/2 | LAMP 1/2 |
|  | Matrix metalloproteinase-9 (type IV collagenase) | MMP-9 |
|  | Not attached to protein | N.a.t.p. |
|  | Plasminogen | PLG |
|  | Platelet glycoprotein Ib alpha chain (glycocalicin) | GPIbA |
|  | Prothrombin | F2 |
|  | P-selectin glycoprotein ligand | PSGL-1 |
|  | Receptor-type tyrosine-protein phosphatase C | CD45 |
|  | Serotransferrin | TF |
|  | Serum amyloid P-component | SAP |
|  | Sex hormone-binding globulin | SHBG |
|  | Solute carrier family 2, facilitated glucose transporter member 1 | GLUT-1 |
|  | Tumor-necrosis factor | TNF-a |
|  | Vitamin D-binding protein | DBP |
|  | Von Willebrand factor | vWF |
| Immune system | Immunoglobulin gamma | Ig g |
|  | Leukosialin | CD43 |
|  | Nonsecretory ribonuclease | Rnase 2 |
|  | Not attached to protein | N.a.t.p. |
|  | Proactivator polypeptide (Prosaposin) | PSAP |
| Integumentary system | Interstitial collagenase | MMP-1 |
|  | Tissue-type plasminogen activator | t-PA |
| Musculoskeletal system | Osteopontin | SPP-1 |
| Nervous system | Amyloid beta A4 | APP |
|  | Not attached to protein | N.a.t.p. |
|  | Serotransferrin | TF |
| Respiratory system | Leukosialin | CD43 |
|  | Mucin | MUC |
|  | Not attached to protein | N.a.t.p. |
| Sensory system | Interphotoreceptor matrix proteoglycan 1 | SPACR |
|  | Rhodopsin | Opsin-2 |
| Urogenital system | Alpha-fetoprotein | AFP |
|  | Alpha-N-acetylgalactosaminidase | NAGA |
|  | Arylsulfatase A | ASA |
|  | Beta-secretase 1 | BACE1 |
|  | Cadherin-5 | CD144 |
|  | CD40 ligand (tumor necrosis factor ligand superfamily member 5) | CD40-L |
|  | CD59 glycoprotein | CD59 |
|  | Chimeric plasminogen activator | K2tu-PA |
|  | Choriogonadotropin subunit alpha and beta | CG-a/b |
|  | Choriogonadotropin subunit alpha | CG-a |
|  | Choriogonadotropin subunit beta | CG-b |
|  | Chromogranin-A | CgA |
|  | Coagulation factor VIII | F8C |
|  | Epidermal growth factor receptor | EGFR |
|  | Erythropoietin | EPO |
|  | Glycodelin | GD |
|  | Glycopeptides | GP |
|  | Glycoprotein hormones alpha chain | FSH-a |
|  | Glycoprotein hormones beta chain | FSH-b |
|  | Glycoprotein LN | GPLN |
|  | Glycoprotein RG | GPRG |
|  | Hepatocyte growth factor | HGF |
|  | Immunoglobulin gamma-1 | Ig g-1 |
|  | Immunoglobulin gamma-3 | Ig g-3 |
|  | Immunoglobulin lambda chain SM | Ig l-SM |
|  | Integrin alpha-5/beta-1 | CD49e/CD29 |
|  | Interferon gamma | IFN-g |
|  | Interferon gamma receptor 1 | IFN-g-R1 |
|  | Interferon omega-1 | IFNW1 |
|  | Interleukin-2 | IL-2 |
|  | Intestinal-type alkaline phosphatase | IAP |
|  | Kallikrein-1 | KLK-1 |
|  | Lactotransferrin | LTF |
|  | Latent-transforming growth factor beta-binding protein 1 | LTBP-1 |
|  | L-selectin | SELL |
|  | Lymphotoxin-alpha | LT-a |
|  | Metalloproteinase inhibitor 1 | TIMP-1 |
|  | Mucin | MUC |
|  | Mucin-1 | MUC-1 |
|  | Next to BRCA1 gene 1 protein | NBR1 |
|  | Not attached to protein | N.a.t.p. |
|  | Phosphatidylcholine-sterol acyltransferase | LCAT |
|  | Plasminogen | PLG |
|  | Renin | REN |
|  | Serotransferrin | TF |
|  | Sialic acid-binding Ig-like lectin 5 | Siglec-5 |
|  | Sialic acid-binding Ig-like lectin 7 | Siglec-7 |
|  | Sialic acid-binding Ig-like lectin 8 | Siglec-8 |
|  | T-cell surface antigen CD2 | CD2 |
|  | T-cell surface glycoprotein CD4 | CD4 |
|  | T-cell surface glycoprotein CD5 | CD5 |
|  | Thrombopoietin | THPO |
|  | Tissue factor pathway inhibitor | TFPI |
|  | Tissue-type plasminogen activator | t-PA |
|  | Transferrin receptor protein 1 | TR |
|  | Uromodulin | UMOD |
|  | Vitamin K-dependent protein C | PROC |
|  | Zona pellucida sperm-binding proteins 3 and 4 | ZP-3/ZP-4 |
| Unknown system | Alpha-fetoprotein | AFP |
|  | Fibronectin | FN |
|  | Immunoglobulin mu | Ig m |
|  | Immunoglobulin M | Ig M |
|  | Myeloperoxisase | MPO |
|  | Not attached to protein | N.a.t.p. |
|  | Serum amyloid P-component | SAP |
|  | Serotransferrin | TF |
